# Bridging micro and macroevolution: insights from chromosomal dynamics in plants

**DOI:** 10.3389/fpls.2025.1606450

**Published:** 2025-08-22

**Authors:** Carmen Benítez-Benítez, Ashwini V. Mohan, Rogelio Sánchez-Villegas, Inés Gómez-Ramos, Ana Valdés-Florido, Kay Lucek, Marek Slovák, Filip Kolář, Ilia J. Leitch, Modesto Luceño, Isabel Larridon, Angelino Carta, Joan Cuscó-Borràs, Enrique Maguilla, Alegría Montero-Ramírez, Patrick G. Meirmans, Alison Dawn Scott, Santiago Martín-Bravo, Marcial Escudero

**Affiliations:** ^1^ Botany Area, Department of Plant Biology and Ecology, Faculty of Biology, University of Seville, Seville, Spain; ^2^ Biodiversity Genomics Laboratory, Institute of Biology, University of Neuchâtel, Neuchâtel, Switzerland; ^3^ Botany Area, Department of Molecular Biology and Biochemical Engineering, Universidad Pablo de Olavide, Seville, Spain; ^4^ Department of Botany, Faculty of Science, Charles University, Prague, Czechia; ^5^ Department of Microbial and Plant Interactions, Plant Science and Biodiversity Centre, Slovak Academy of Sciences, Institute of Botany, Bratislava, Slovakia; ^6^ Czech Academy of Sciences, Institute of Botany, Průhonice, Czechia; ^7^ Royal Botanic Gardens, Kew, Richmond, United Kingdom; ^8^ Department of Biology, Botany Unit, University of Pisa, Pisa, Italy; ^9^ Centre for Climate Change Impact (CIRSEC), University of Pisa, Pisa, Italy; ^10^ Institute for Biodiversity and Ecosystem Dynamics (IBED), Faculty of Science, University of Amsterdam, Amsterdam, Netherlands; ^11^ Department of Chromosome Biology, Max Planck Institute for Plant Breeding Research, Cologne, Germany

**Keywords:** angiosperms, chromosome, dysploidy, evolution, polyploidy, speciation

## Abstract

Understanding the relationship between macro- and microevolutionary processes and their delimitation remains a challenge. This review focuses on the role of chromosomal rearrangements in plant population differentiation and lineage diversification resulting in speciation, helping bridge the gap between macro- and microevolution through chromosomal evolution. We focus on angiosperms, a group that comprises the majority of extant plant species diversity and exhibits the largest chromosomal and genomic variations. Here, we address the following questions: Are macroevolutionary patterns of chromosome evolution the result of accumulated microevolutionary changes, or do chromosomal dynamics drive larger shifts along the speciation continuum? At the macroevolutionary level, we investigated the association between karyotype diversity and diversification rates using evidence from comparative genomics, chromosomal evolution modelling across phylogenies, and the association with several traits across different angiosperm lineages. At the microevolutionary level, we explore if different karyotypes are linked to morphological changes and population genetic differentiation in the same lineages. Polyploidy (autopolyploidy and allopolyploidy) and dysploidy are known drivers of speciation, with karyotypic differences often leading to reproductive barriers. We found that dysploidy, involving gains and losses of single chromosomes with no significant change in overall content of the genome, appears to be relatively more frequent and persistent across macroevolutionary histories than polyploidy. Additionally, chromosomal rearrangements that do not entail change in chromosome number, such as insertions, deletions, inversions, and duplications of chromosome fragments, as well as translocations between chromosomes, are increasingly recognized for their role in local adaptation and speciation. We argue that there is more evidence linking chromosomal rearrangements with genetic and morphological trait differentiation at microevolutionary scales than at macroevolutionary ones. Our findings highlight the importance of selection across evolutionary scales, where certain chromosomal dynamics become fixed over macroevolutionary time. Consequently, at microevolutionary scales, chromosome rearrangements are frequent and diverse, serving as key drivers of plant diversification and adaptation by providing a pool of variation from which beneficial chromosomal changes can be selected and fixed by evolutionary forces.

## Introduction

### The role of chromosomal alterations in evolution: a brief overview

Understanding the relative importances of different chromosomal dynamics across macro- and microevolutionary processes remains a challenge in the field of evolutionary biology (Lucek et al., 2023). However, research has advanced significantly due to the unprecedented amount and accuracy of genomic datasets being available. This review focuses on chromosomal evolution in plants, especially angiosperms, from both micro- and macroevolution perspectives. It spans processes at the intraspecific level (population dynamics), cladogenetic events (lineage diversification and speciation), to broad patterns observed across large number of species (macroevolution). Specifically, by focusing on angiosperms, a group that comprises the majority of extant plant species diversity (Benton et al., 2021) and exhibiting the largest variation in chromosomal and genomic assembly across living organisms (Escudero and Wendel, 2020), we take into consideration the impact of different chromosomal rearrangements (CRs) in the process of plant population differentiation, lineage diversification and speciation. While recent technological and methodological advances allow the detection of CRs at a much broader scope, their evolutionary implications are still elusive (reviewed in Lucek et al., 2023). While Lucek et al. (2023) reviewed the implications of several types of CRs, many CRs that are especially widespread in plants such as CRs resulting from polyploidization have not been considered. Chromosomal rearrangements associated with diversification (Carta and Escudero, 2023) do not seem to be primarily driven by ecological adaptation, instead, they may arise from mutational processes or intrinsic genetic conflicts (Maheshwari and Barbash, 2011). Particularly, factors that limit the exchange of genetic material are crucial in understanding how chromosomal dynamics can result in adaptation and speciation. Chromosomal rearrangements can reduce gene flow by affecting recombination, but these alone do not fully account for most models of chromosomal speciation (Rieseberg, 2001a). One reason for this is that the restriction of gene flow across a large chromosomal block is often insufficient to drive the speciation process on its own, unless the rearrangement involves key regions that contribute to reproductive isolation. Such key regions can be a single gene or a set of linked genes, depending on the organism, e.g. inversions of crucial chromosomal regions have been shown to drive speciation in plants (Huang and Rieseberg, 2020). However, a synergistic interaction between isolation genes (those contributing to reproductive barriers) and CRs can enhance the likelihood of models of speciation, especially when the CRs are only weakly underdominant (Rieseberg, 2001a). Chromosomal rearrangements can cause problems in chromosome pairing during meiosis, leading to reduced fertility in hybrids by producing unbalanced gametes (Rieseberg, 2001a; Stathos and Fishman, 2014). This reduction acts as a partial reproductive barrier, decreasing the probability of successful gene exchange between populations with different karyotypes and CRs. Thus, while CRs may not always be the primary driver of speciation, their direct or indirect interactions with other reproductive isolating mechanisms could contribute to playing a significant role in the process.

Cytogenetic mutations can alter the number of chromosomes, their composition, the order of the genetic material within them, or interactions between chromosomes. Chromosomal rearrangements may be classified into two main categories: those that lead to a change in chromosome number and those that result in structural changes, i.e. within a chromosome (see **Box 1**). Regarding the former, polyploidy involves the duplication of one or more chromosome sets. From CRs that impact chromosome number, polyploidy has received the most attention, even though chromosome gains and losses are highly prevalent among angiosperms (Grant, 1981; Otto and Whitton, 2000; Rice et al., 2015; Stebbins, 1971). In particular, whole genome duplication (WGD) plays an important role in plant evolution at different temporal scales, with profound effects from molecular to ecological levels (Stebbins, 1971; Grant, 1981), including the restoration of fertility after hybridization (Charron et al., 2019). This phenomenon also has significant effects on gene expression, often resulting in epigenetically induced gene silencing (Osborn et al., 2003). It is particularly important, as many of the world’s crops (especially those essential to global food production, such as wheat (*Triticum aestivum* L.), maize (*Zea mays* L.) and potato (*Solanum tuberosum* L.)) have a polyploid origin (Adams and Wendel, 2005; Tate et al., 2005)

Polyploids can also return back to a diploid state over time through diploidization, which comprise a diverse set of molecular processes leading to gene losses, genome downsizing and chromosomal fusions (Mandáková and Lysak, 2018). These processes can facilitate the stabilization of polyploids and enhance their ability to adapt to new environments. Such processes have potential implications for the evolutionary history of species (e.g., distribution patterns, life history and ecological adaptations). In angiosperms, diploidization may lead to species with relatively low chromosome numbers and small genome sizes (Luo et al., 2009; Mandáková et al., 2010a). While many plants are classified as diploids, the increasing amounts of sequenced genomes data are now revealing that many are diploidized polyploids. In fact, it is now recognized that all angiosperms have undergone at least one polyploidy event (often two or more) during their evolutionary history (Jiao et al., 2011; Van de Peer et al., 2017; Chen et al., 2024). Although this was not definitively proven cytogenetically or from a microevolutionary point of view, it is apparent from a macroevolutionary perspective that diploidization has played a crucial role in the evolution of vascular plants, particularly in angiosperms, as seen in model cases such as Brassicaceae (Mandáková et al., 2016; Mandáková and Lysak, 2018).

Apart from polyploidy, another kind of CR causing changes in chromosome number is dysploidy (Guerra, 2016). This mechanism produces changes in the karyotype configuration but results in no significant changes in DNA content (Heilborn, 1924; Luceño and Guerra, 1996; Escudero et al., 2014). This is especially frequent in plants with holocentric chromosomes, i.e. chromosomes that lack a single centromere but have centromeric regions spread across their chromosomes (Da Silva et al., 2017; Escudero et al., 2018; Márquez-Corro et al., 2019; Johnen et al., 2020). However, the potential impact of dysploidy on rates of diversification (henceforth encompassing both speciation and extinction processes) has not been studied in detail because it is challenging to link chromosome number evolution and species diversification at a macroevolutionary scale (but see Tribble et al., 2025 for an example of association between dysploidy and diversification rates). Interestingly, karyotype changes arising from dysploidy are thought to persist longer over time than those from polyploidy (Escudero et al., 2014; Sader et al., 2019).

Furthermore, there are CRs that do not entail changes in chromosome number, but imply structural changes. These structural variants (SVs) are known to cause chromosomal disorders, affecting mainly the phenotype and size of chromosomes (Lysak and Schubert, 2012; see more details in **Box 1**). A large amount of SVs has been observed at the whole-genome level between individuals belonging to related species (Lysak et al., 2006; Seymour et al., 2014). However, recurrent rearrangements with similar breakpoints, sizes and genomic context can also be shared by unrelated individuals (Mandáková and Lysak, 2008; Carvalho and Lupski, 2016).

### Box 1. Chromosomal rearrangements entailing change in chromosome number vs. structural changes within a chromosome

**Figure 1:**
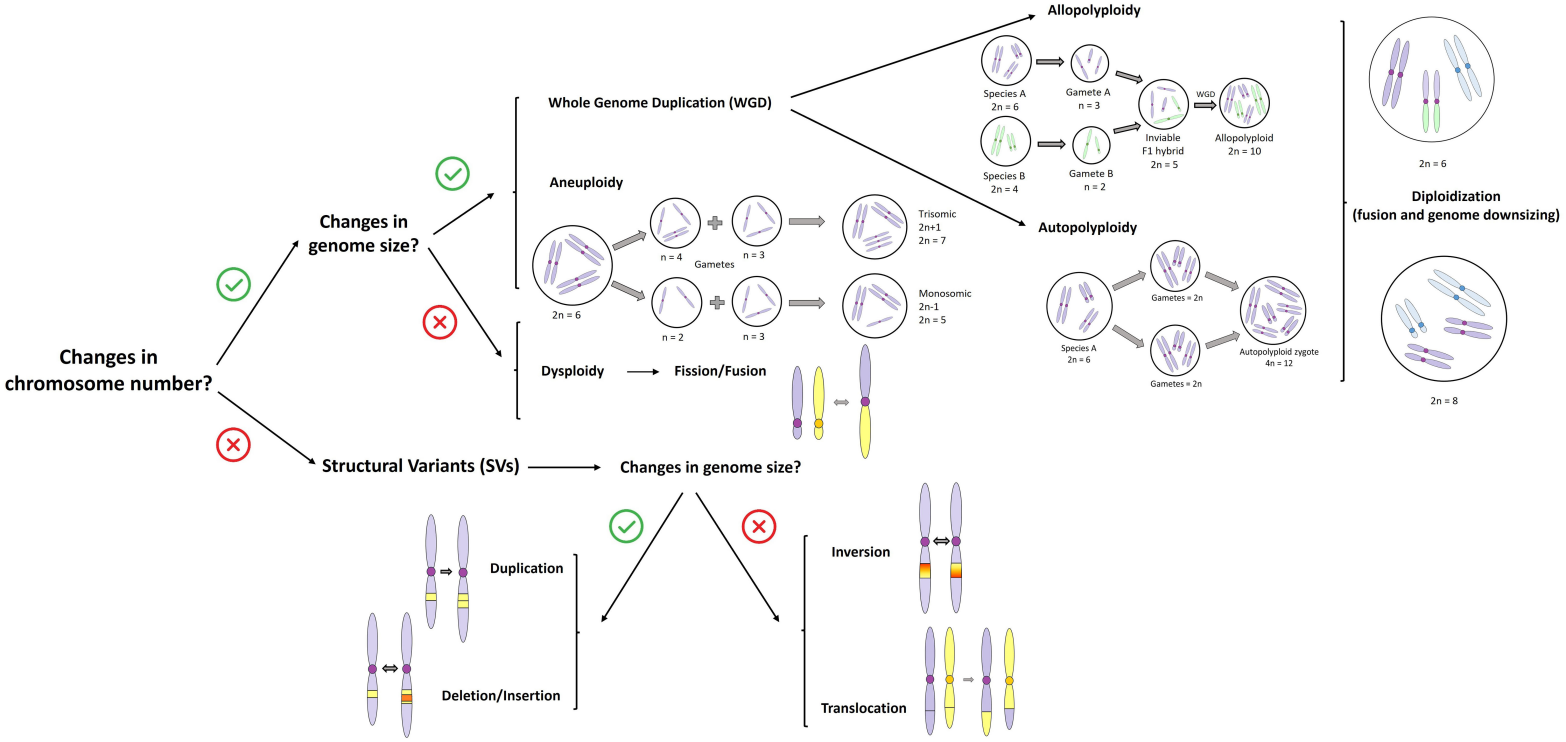
Summary of the main types of chromosomal rearrangements (CRs) that do (or not) entail changes in chromosome number (>50bp following Berdan et al., 2024). The figure shows the main pathway within allopolyploidy to produce a polyploid hybrid. For more cases of allopolyploid hybridization see in detail Hegarty and Hiscock (2008). The blue color in chromosomes indicates complex rearrangements that result in genome size reduction in the diploidization process.

A whole genome duplication (WGD) event entails abrupt multiplication of chromosomal sets, resulting in a polyploid individual. This process can generally be categorized into two types, although in reality, there is a continuum between them (**Figure 1**): (i) autopolyploidy, which involves the multiplication of chromosomes within the same species, usually involving the fusion of one or two unreduced gametes, and which may result in rapid reproductive isolation between individuals with different ploidy levels (Stebbins, 1947; Soltis and Soltis, 1989; Servick et al., 2015), and (ii) allopolyploidy, which involves the combination of chromosome sets from different parental species via hybridization between different species followed by polyploidy (Barrier et al., 1999; Rodionov, 2023; Van der Heijden et al., 2024). Autopolyploidy is very common in plants (Parisod et al., 2010; Scarrow et al., 2020) but has received less attention compared to allopolyploidy. This is because it was long believed to have little impact on plant divergence due to its formation through genome duplication only, without the involvement of hybridization. However, other advances in plant molecular biology suggest that both auto- and allopolyploidy have significant roles for evolutionary adaptation and subsequent divergence of plant species (Barker et al., 2016). Autopolyploidy, allopolyploidy, and dysploidy are the most important CRs recognized in the evolutionary history of plants (Mandáková and Lysak, 2018; Mandáková et al., 2018).

Dysploidy involves gains (ascending dysploidy via chromosome fission) or losses (descending dysploidy via chromosome fusion) of single chromosomes (Mayrose et al., 2010; Escudero et al., 2014) and appears to be relatively frequent and with longer persistence during evolutionary history than polyploidy (Escudero et al., 2014; Carta et al., 2020). While aneuploidy involves chromosome duplications and losses that result in changes in DNA content (**Figure 1**), such a condition is strongly selected against and tends to have little evolutionary persistence (Escudero et al., 2014). Diploidization is the process of converting a polyploid back into a diploid one. This is also a significant process in the evolutionary history of polyploids, as it can facilitate their adaptation and environmental establishment (Zhong et al., 2022; Huang et al., 2023), thereby contributing to their evolutionary success by eliminating redundant genetic material or resolving meiotic irregularities (**Figure 1**).

Otherwise, structural variants can arise from various molecular mechanisms, such as DNA replication, DNA repair, and recombination processes. These processes can give rise to duplications or deletions of chromosome fragments, as well as translocations and, especially, inversions. While the two latter typically do not involve gain or loss of genetic material, they do rearrange gene order along the chromosome (**Figure 1**). Despite this, such rearrangements might still disrupt coding or regulatory sequences and alter chromatin structure. Consequently, they provide a mechanistic basis for how CRs may act as drivers of evolution.

### Methods and models to study chromosomal rearrangements

Several methodologies are available to study chromosome number evolution, as well as CRs and their breakpoints. The earliest approach for investigating changes in chromosome number involved optical cytogenetic techniques to count chromosomes from mitotic or meiotic cells and karyotype them (Guerra, 2008). Many genomic disorders caused by SVs were initially uncovered by these early cytogenetic methods. For instance, the classical protocol for detecting inversions were based on observations of the strength of linkage of hybrids between different strains, showing an inverted order of genes with respect to a reference strain (Sturtevant, 1921; Dobzhansky and Sturtevant, 1938).

Most recently, the progress in molecular cytogenetic techniques combined with high throughput DNA sequencing, has enabled the rapid and precise detection of CRs across the genome at increasingly high levels of resolution. Such results are now increasingly being combined with insights gained from chromosome level whole genome assemblies to reveal the nature of the DNA sequences including single nucleotide polymorphisms (SNPs) associated with CRs (Le Scouarnec and Gribble, 2012). More advanced cytogenetic techniques based on *in situ* hybridization methods, such as FISH (fluorescence *in situ* hybridization) and GISH (genomic *in situ* hybridization), are widely used and effective for investigating chromosomal evolution. These methods enable us to visualize CRs as well as changes in chromosome number introduced through aneuploidy, dysploidy and polyploidy (Chester et al., 2010; Jiang, 2019).

The era of whole genome sequencing has revolutionized the ability to detect genomic rearrangements with unprecedented precision. By utilizing the order and spacing of genomic regions, these rearrangements can be quantified through synteny analyses, providing ever deeper insights into genome evolution and structure (Tang et al., 2008). Among the latest computational innovations, tools like SyRI (Goel et al., 2019) allow the detailed identification of CRs by comparing whole-genome assemblies. These results are visualized using Plotsr implemented as a Python package (Goel and Schneeberger, 2022), a tool that graphically represents synteny and rearrangement patterns across genomes, enabling the exploration of structural differences.

Complementing these genomic approaches, the combination of karyotypic and cytogenetic data (chromosome number and DNA content) is being examined within a phylogenetic framework that accounts for non-independence using comparative methods and models of chromosome evolution (Costa et al., 2017). The use of probabilistic models such as ChromEvol (Mayrose et al., 2010; Glick and Mayrose, 2014) that allow inferences of chromosome number changes across molecular phylogenies is becoming more frequent. More recently, the development of comparative phylogenetic frameworks, i.e. ChromoSSE (Freyman and Höhna, 2018), further enables researchers to determine the degree to which CRs, including chromosomal fusions, fissions and WGD, are anagenetic or cladogenetic (in this second case there is an association between CRs and diversification). Modern comparative phylogenetic methods, and more specifically models developed to study chromosome number variation across phylogenetic trees together are increasingly enabling us to uncover the key roles played by karyotypic changes in the evolution of plants.

### Chromosomal rearrangements and their role in the diversification of land plants

The study of plant evolution has long focused on understanding polyploidy and CRs, both of which play an important role in shaping genome structure. While angiosperms have been extensively studied in this context, other vascular plant groups non-commercially important as food crops, such as gymnosperms or ferns, are now gaining attention due to recent technical development and the declining cost of DNA-sequencing, which have made genome data more widely available. Advances in sequencing technologies and analytical pipelines have provided valuable insights into genome size evolution and the mechanisms driving their evolution.

Genomic studies have shown that angiosperm evolution is rich in WGD events (Paterson et al., 2012), with each subsequent polyploid event layered upon the genomic remnants of earlier rounds of polyploidization events (Wendel, 2015; Carta et al., 2020; Escudero and Wendel, 2020). Thus, chromosome doubling has played an important role in the diversification of many genera of angiosperms (Wendel et al., 2018; Barker et al., 2024). Studies on fern genomes have highlighted that recurrent WGD events without subsequent diploidization and reduction in genome size may explain several key genomic characteristics (Clark et al., 2016; Kinosian and Barker, 2024). In contrast to angiosperms, ferns exhibit a significantly higher number of chromosomes, likely driven by a greater number of meiotic events, which contributes to increased rates of polyploid speciation (Klekowski and Baker, 1966; Carta et al., 2020; Zheng et al., 2024). This suggests that the diploidization process following polyploidy is less strong or prevalent in ferns, for which the average rate of chromosome loss is estimated to be about half the rate of angiosperms (Zheng et al., 2024). Thus, this lower estimated rate of chromosome loss among ferns is consistent with their typically higher number of chromosomes, compared with angiosperms (Klekowski and Baker, 1966; Carta et al., 2020; Zheng et al., 2024). In addition, and in contrast to angiosperms and gymnosperms, a clear positive correlation between genome size and chromosome number has also been found in ferns with larger genomes having more chromosomes (Barker, 2013; Leitch and Leitch, 2013).

Genomic and chromosomal phylogenetic analyses have shown that angiosperms have the highest rates of ancient WGD and dysploidy among vascular plants, while ferns seem to experience multiple rounds of polyploid speciation events followed by gene silencing but not chromosome losses (Haufler, 1987; Zheng et al., 2024). Particularly, genome size reconstruction studies across angiosperms suggest that their ancestral genome size was very small compared to their plant relatives, gymnosperms and ferns (Leitch and Bennett, 1997, 2002; Soltis et al., 2013; Clark et al., 2016; Pellicer et al., 2018). This pattern is consistent across most major clades of flowering plants, including both monocots and eudicots (Leitch and Bennett, 1997; Wendel, 2015; Escudero and Wendel, 2020). Although angiosperms and gymnosperms may be subject to similar selection pressures for genome size reduction (e.g., nutrient limitations, drought stress), only angiosperms appear to have the molecular mechanisms necessary to achieve significant decreases in genome size (Michael, 2014; Ezoe and Seki, 2024).

The evidence from the ancestral node shared between angiosperms and gymnosperms suggests that genome duplication did not occur during the initial emergence of angiosperms but may have happened later (Wendel, 2015; Carta et al., 2020; Escudero and Wendel, 2020). Within angiosperms, monocots exhibit a clear pattern of repeated genome duplication throughout the diversification of various genera, while the common ancestor of eudicots is considered to have undergone a whole genome triplication (Jiao et al., 2012). Polyploidy has also recurred in many lineages that have diversified more recently within this group (e.g., *Brassica*, *Solanum*; Wendel, 2015; and at the base of many families including Asteraceae, Fabaceae, Brassicaceae, Ranunculaceae; Qiao et al., 2019). These cycles of polyploidy tend to repeat over timescales ranging from thousands to millions of years (Wendel, 2015; Escudero and Wendel, 2020). As outlined above, polyploidy is, at least in part, reversible. Over time, it is often followed by extensive CRs, reductions in chromosome number, and large-scale losses of both repetitive sequences and duplicated genes, ultimately leading to genome downsizing (Leitch and Leitch, 2008). This diploidization phenomenon involves diverse processes that result in descendants behaving cytogenetically as typical diploids, while still retaining vestigial evidence of past polyploidy events within their genomes (Wendel, 2015; Carta et al., 2020; Escudero and Wendel, 2020). In addition, during diploidization, one of the two genomes is preferentially retained and exhibits higher gene expression levels, as widely observed across angiosperm lineages (Cheng et al., 2012; Wendel, 2015).

Despite the advances reported above, our understanding of CRs in the process of plant population differentiation and lineage diversification in ferns and gymnosperms remains more limited, not allowing a detailed overview as for angiosperms. In light of this, our review provides new insights into the mechanisms underlying the transition from macro- to microevolutionary processes focusing on angiosperms, contributing to a deeper understanding of evolutionary dynamics across both scales. To this end, we addressed six questions (Q1-Q6) allowing us to present the topic in a systematic way. Specifically, at the macroevolutionary level, we investigate the association between karyotype diversity and rates of diversification, discussing comparative genomics and chromosomal evolution modeling across phylogenies (Q1-Q2). We also explore how CRs correlate with several traits across different angiosperm lineages (Q3). At the microevolutionary level, we examine how different karyotypes (including differences in ploidy level, chromosome number and structure) are linked to geographic, environmental, and phenotypic changes (including anatomical and physiological shifts; Q4-Q5). Additionally, we investigated how population genetic differentiation through allo- and autopolyploidy may promote the formation of new genetic combinations (Q6). Understanding the evolutionary processes leading to intraspecific chromosomal diversification is crucial, as such divergence can lead not only to reproductive incompatibilities between species but also to diversification and speciation processes within a given species. Ultimately, the goal of this review is to address whether patterns of chromosomal macroevolution reported in the literature are the result of singular chromosomal changes accumulating within microevolutionary timeframes, or alternatively, whether some chromosomal changes are able to cause a larger shift in the speciation continuum compared to others.

## Q1: How have patterns of CRs shaped the recent evolutionary history of angiosperms?

Understanding how evolutionary trends have changed through time is challenging, as it involves the interaction of several factors. The most important among these challenges are the ability to distinguish true evolutionary patterns (actual evolutionary events) from those influenced by sampling bias towards the present (i.e., only extant representatives of diverse clades) and the unequal extinction rates across different groups of plants.

The macroevolutionary study of chromosomal changes through evolutionary history utilizing comparative phylogenetic frameworks is no different. These studies focus on karyotypic changes that lead to changes in chromosome number, through poly- and dysploidy, as chromosome counts are widely available for many plants (see **Figure 1** in Carta and Escudero, 2023; The Index to Plant Chromosome Numbers: Goldblatt and Johnson, 2011; Goldblatt and Lowry, 2011; The Chromosome Counts Database: Rice et al., 2015; Rice and Mayrose, 2023). In contrast, genomic or detailed karyotypic data needed for detecting other types of CRs, such as inversions or translocations, have only started to be generated in the last two to three decades.

The two major processes underpinning chromosome losses and gains (i.e. aneuploidy and dysploidy) have different impacts on lineage diversification. While aneuploidy leads to unstable lineages that persist for shorter periods over evolutionary time (under 1–5 million years; Yona et al., 2012), dysploidy has a less drastic impact, resulting in more stable lineages throughout time (Otto, 2007). Most, if not all, of the chromosome gains and losses that have been inferred in phylogenies of angiosperms are caused by dysploidy (Escudero et al., 2014; Carta et al., 2020). For instance, in the genus *Carex* L. (Cyperaceae), one of the most diverse genera of vascular plants in terms of species richness and chromosome number diversity, there is a tendency for dysploidy to be the main underlying mechanism responsible for chromosome evolution (Hipp et al., 2009).

**Figure 2:**
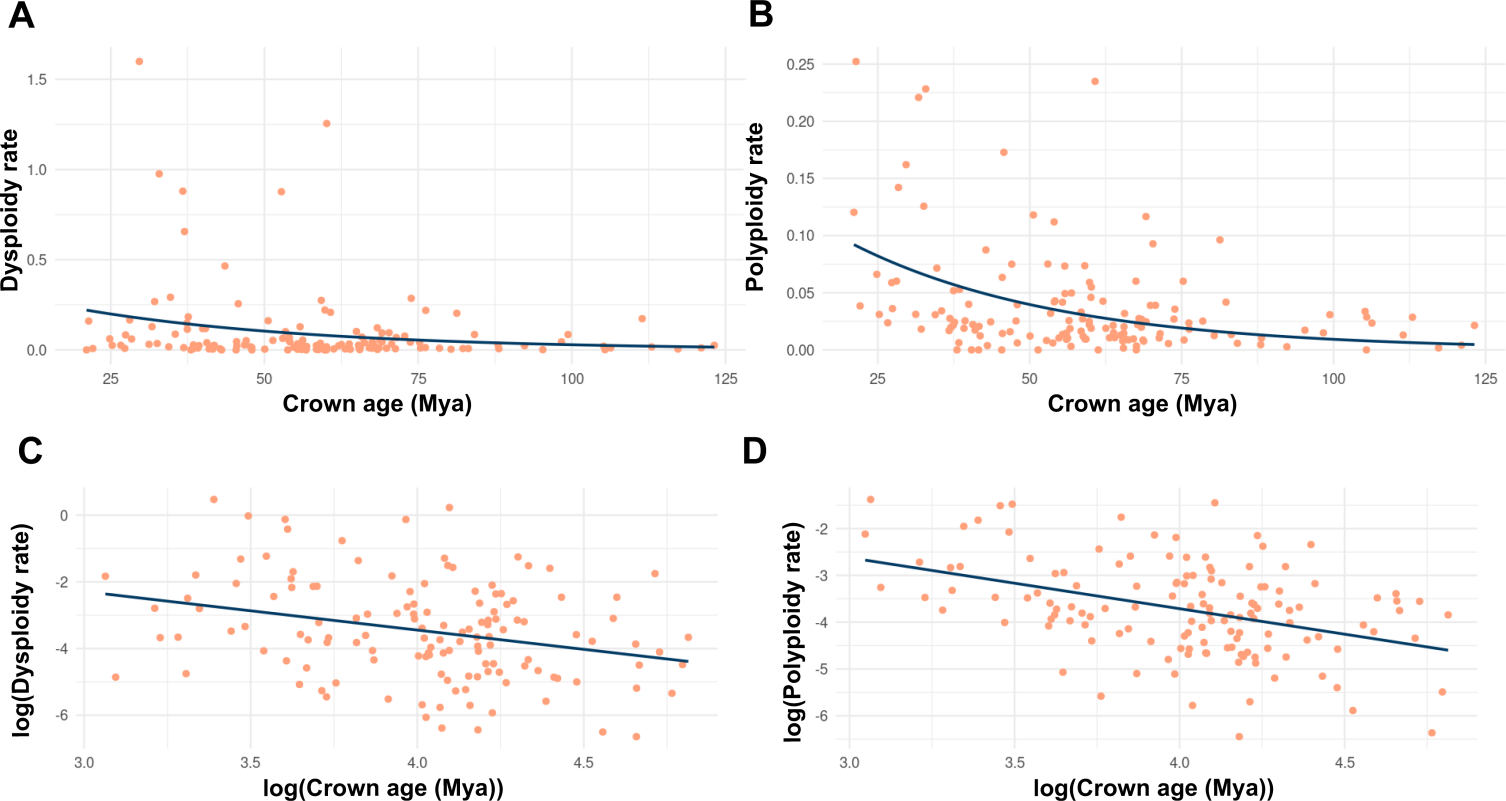
Timescale-dependent patterns of chromosome rearrangements, based on phylogenetic and chromosome count data from Zhan et al. (2021), who inferred the relationships between polyploidy, dysploidy with lineage diversification by combining chromosome number data with a time-calibrated mega-phylogeny, assembling clade-level datasets for 30,000 taxa representing 46 orders and 147 families of angiosperms. Each dot represents a clade for which polyploidy and dysploidy rates were estimated using ChromEvol. **(A, C)** for dysploidy, **(B, D)** for polyploidy, **(A, B)** for non-transformed data and **(C, D)** for log-transformed data.

**Figure 3:**
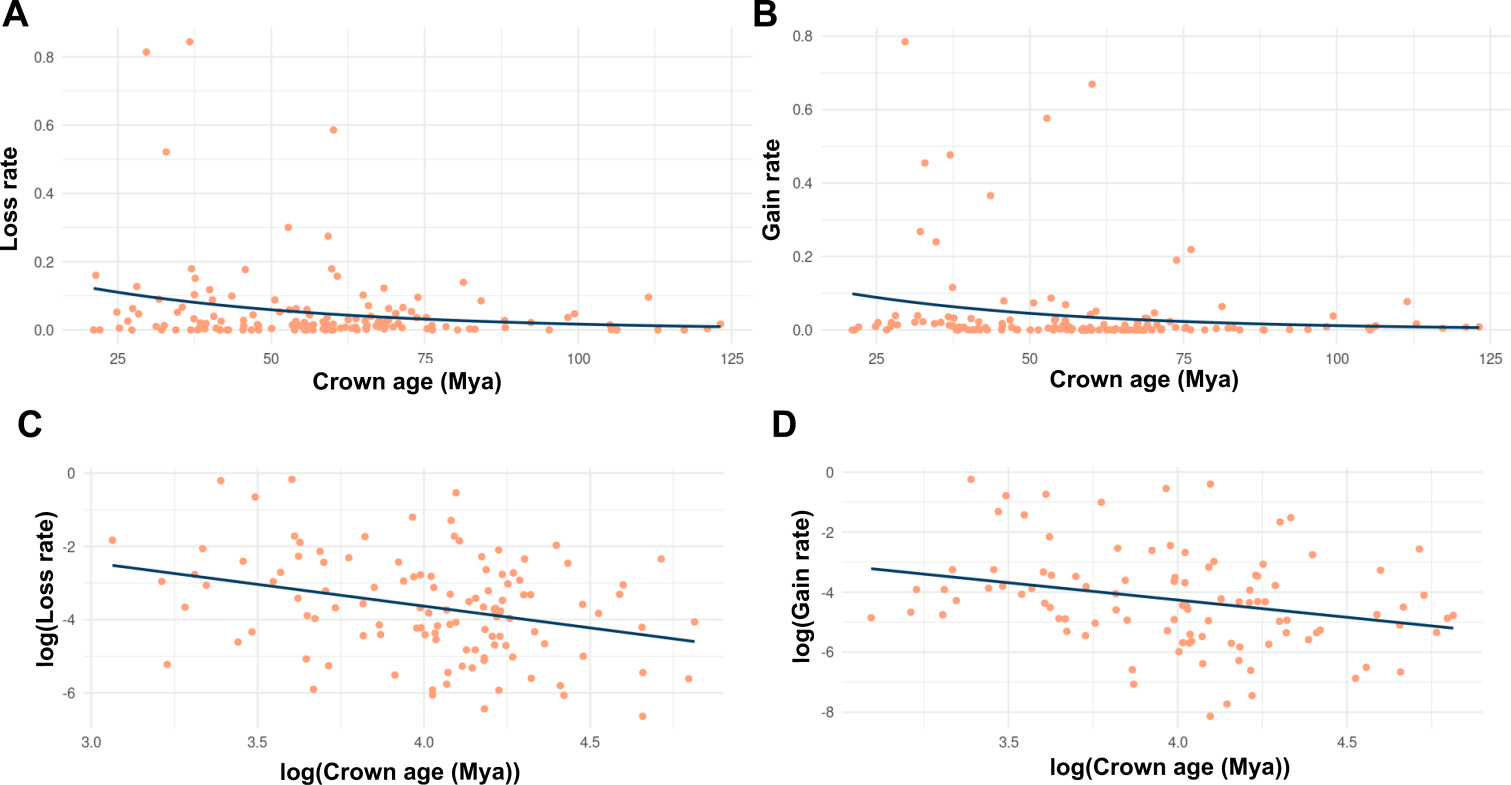
Timescale-dependent patterns of chromosome rearrangements based on phylogenetic and chromosome count data from Zhan et al. (2021), who inferred the relationships between descending (chromosome loss) or ascending (chromosome gain) dysploidy with lineage diversification by combining chromosome number data with a time-calibrated mega-phylogeny, assembling clade-level datasets for 30,000 taxa representing 46 orders and 147 families of angiosperms. Each dot represents a clade for which descending or ascending dysploidy rates were estimated using ChromEvol. **(A, C)** for descending dysploidy (chromosome loss), **(B, D)** for ascending dysploidy (chromosome gain), **(A, B)** for non-transformed data and **(C, D)** for log-transformed data.

Multiple studies have found that rates of polyploidy have increased in more recent evolutionary time in angiosperms (Carta et al., 2020; Escudero et al., 2014; see in **Figure 2** data from Zhan et al., 2021). In the case of dysploidy, the rates are more constant and slightly accelerate towards the present (Escudero et al., 2014; see in **Figure 2**). Data suggest that there are no significant differences between descending and ascending dysploidy rates (**Figure 3**). Here, we show the rate of dysploidy and polyploidy against time using the data from Zhan et al. (2021), who inferred rates of polyploidy and dysploidy using ChromEvol (Glick and Mayrose, 2014; Mayrose et al., 2010) from phylogenetic (Qian and Jin, 2016; Zanne et al., 2014) and chromosome count data (Rice et al., 2015) for 30,000 taxa representing 46 orders and 147 families of angiosperms. Their analyses show that both polyploidy and dysploidy increase exponentially with time, but the increase in rate is much greater for polyploidy (**Figure 2**). The evolution of CRs through time is yet to be explored for gymnosperms and ferns, which could be especially interesting in the latter as they have an even greater chromosome number variation than angiosperms (Leitch and Leitch, 2012) and similar rates of paleopolyploidy (Li et al., 2024).

This acceleration towards the present in CRs is probably partially caused by a detection bias, as reconstructing genomes further back in time requires increasingly extensive genomic data to remain accurate. Chromosome rearrangement inference from chromosome counts is less reliable with increasing phylogenetic depth as homoplasies become more frequent, especially within groups with high karyological instability (Mayrose and Lysak, 2021; Weiss-Schneeweiss and Schneeweiss, 2013). Methods based on either genetic (gene duplicate analysis and paralogue divergence) or genomic data (synteny analysis), are more reliable at higher depths than those based on chromosome counts, even allowing detection of other CRs (Wang et al., 2021). As more genomic data become available for a wider range of taxa, studies of CR events in angiosperm history will become more accurate. One of the most recent studies addressing this issue was performed by McKibben et al. (2024). The authors studied synonymous divergences of paralogs (Ks) and orthologs, along with syntenic analysis on 462 genomes distributed throughout the angiosperm phylogenetic tree. Their analyses inferred multiple ancient WGD events and concluded that most extant species have experienced, on average, three WGD events, while also detected an increase in WGD towards the tips of the phylogenetic tree.

While a bias towards more recent events may contribute to the apparent acceleration in rates of CR, it does not account for the higher pattern of acceleration observed in polyploids compared to dysploids (**Figure 2**). This bias would be expected to influence both types of CRs, and potentially even more strongly for polyploidy because it is generally easier to detect than dysploidy. Thus, the discrepancy in acceleration rates suggests additional factors are at play. One hypothesis shows that the detection of polyploidization in the past is more difficult because they stay “polyploid” for a shorter time due to the activity of diploidization processes leading to the rearrangement of multiple sets of parental chromosomes in their polyploid ancestors (Zhang et al., 2019). Considering the amount of WGD throughout the evolution of angiosperms (McKibben et al., 2024), extant species would be expected to have a much higher number of chromosomes and larger genomes than what is currently observed. This discrepancy underscores the impact of diploidization in shaping angiosperm genomes (Wang et al., 2021). Most species undergo a process of diploidization after WGD, which impacts at a genomic, epigenomic and proteomic level (Dodsworth et al., 2016; Wendel et al., 2016). This process often results in a reduction in genome size and a decrease in chromosome number through dysploidy, sometimes reaching a number of chromosomes equal, or even lower, than the original diploid number (Li et al., 2021; Mandáková and Lysak, 2018). Different factors have been proposed to explain the so-called “large genome constraint hypothesis”, which is primarily linked to genome size. These include limitations in phosphorus (P) and nitrogen (N) availability, constraints related to life cycle duration, narrow ecological tolerances, or small population sizes (Knight et al., 2005; Carta and Peruzzi, 2016; Guignard et al., 2016; Wang et al., 2021). Also, there is strong evidence that supports a lower diversification rate for neopolyploids in both ferns and angiosperms (Mayrose et al., 2010). Diploidization mechanisms remain to be studied in gymnosperms and ferns (Li et al., 2021) in order to understand how it impacts CR rates. A study in the hexaploid coast redwood (*Sequoia sempervirens*), one of the few polyploid species in conifers (see other examples in Farhat et al., 2019; 2022), showed very low diploidization rates (Scott et al., 2016). This finding may help to explain the evolutionary success of polyploid lineages and a limited chromosome number variation in gymnosperms. Ferns propose a more interesting, yet unexplored, study system for the impact of diploidization on CR rates. Unlike angiosperms, ferns undergo diploidization primarily through gene deletion and pseudogenization rather than gene loss (Haufler, 1987; Zheng et al., 2024), while still maintaining a high rate of diploidization (Haufler and Soltis, 1986; Haufler, 1987; Wolf et al., 1987).

Descending dysploidy is one of the most frequent routes of diploidization contributing to the reversion towards functional diploidy of polyploid angiosperms (Mandáková and Lysak, 2018). Therefore, descending dysploidy is expected to accelerate after WGD, while this acceleration is not present in species undergoing increasing dysploidy. The latter is often driven by chromosomal fragmentation but does not directly impact genome size significantly, unless accompanied by changes in DNA content. To differentiate the decreasing dysploidy associated with diploidization, we compared decreasing and increasing dysploidy rates to test if they were significantly different (**Figure 3**). However, we did not observe a difference, suggesting that the decreasing dysploidy associated with subsequent diploidization is not as easily detected as ancient WGD (**Figure 2**). This suggests that ancient WGD and the associated chromosome number reductions (decreasing dysploidy) during diploidization are difficult to detect, as polyploids may undergo rapid diploidization, which may limit their diversification potential in the polyploid state. Further support for this comes from models predicting 3–4 rounds of WGD in angiosperms, with high rates of diploidization alongside lower polyploid diversification rates compared to diploids (Barker et al., 2016; 2024). This instability is not observed following dysploidy, as its rate appears to remain more constant throughout angiosperm evolutionary time (Escudero et al., 2014; **Figure 2**). Such findings support the hypothesis that dysploidy, unlike polyploidy, is not as disadvantageous in generating long-term persisting lineages and does not entail significant changes in DNA content (Escudero et al., 2014). Overall, this highlights the importance of further exploring the impact of dysploidy in evolution, which has traditionally received less attention compared to polyploidy, despite its potential influence on chromosomal evolution and species diversification at both macro- and microevolutionary scales. Even less is known about the role of dysploidy in other plant lineages. Descending dysploidy associated with diploidization has been demonstrated to be lower in ferns than in angiosperms even for similar rates of paleoploidy (Haufler, 1987; Li et al., 2024; Zheng et al., 2024), leading to high chromosome numbers (Leitch and Leitch, 2012).

In conclusion, the increase in CR rates in more recent angiosperms is influenced by both biological processes and methodological biases. While sampling biases favor the detection of recent chromosomal changes, true evolutionary mechanisms, particularly polyploidy, also contribute to this tendency. Polyploidy rates increase more rapidly than dysploidy, where ancient WGD followed by diploidization are common. Dysploidy, while more constant over time, shows a slight acceleration toward the present. Models like ChromoSSE have been developed to reconstruct chromosomal evolution by integrating chromosomal changes, speciation, and extinction rates. These models offer valuable insights into how CRs, such as dysploidy and polyploidy, influence diversification across lineages. However, the detection of these patterns is greatly affected by the availability of data (phylogenetic and chromosome number data). Incorporating more comprehensive datasets in future studies, not only within angiosperms but expanding to the rest of plants, may provide a clearer understanding of the great differences between the main plant groups. Further studies should focus on chromosome evolution in ferns, in order to understand how the differences in diploidization mechanisms have shaped chromosome evolution in ferns and angiosperms, both groups with high polyploidy rates.

## Q2: Do chromosome changes driving diversification occur prior to cladogenesis?

Chromosomal rearrangements have the potential to drive speciation by reducing gene flow between divergent populations (Berdan et al., 2024; Lucek et al., 2023). Specifically, chromosomal changes may occur at the speciation event, either by directly initiating a cladogenetic process or by reinforcing speciation that was already initiated or completed by other geographical or ecological drivers or other genomic mutations independent of CR. Allopolyploid speciation is an example of chromosomal changes and cladogenesis coinciding (see section related to allopolyploidy). Currently, two primary models explain how CRs may contribute to reproductive isolation and speciation (Rieseberg, 2001a): hybrid dysfunction and the suppression of recombination.

The classical models are based on hybrid dysfunction and hypothesize that hybrids resulting from crosses between two different chromosomal races have reduced fitness (Coyne and Orr, 2004). This reduced fitness can lead to strong selection against hybrids, primarily because newly arising CRs are often underdominant. While strongly underdominant rearrangements are unlikely to reach fixation, those with weaker underdominance may become fixed but usually create only shallow barriers to reproductive isolation, making them unlikely to drive speciation (Rieseberg, 2001a; Faria and Navarro, 2010). This model of chromosomal speciation suggests that chromosomal changes occur at, or just prior to a cladogenetic event, as such changes are expected to establish significant barriers to gene flow that may result in speciation. In plants, ploidy changes are generally expected to lead to hybrid dysfunction (Escudero et al., 2016b; 2024) because for example, a cross between a diploid and a tetraploid typically produces an inviable or sterile triploid (Köhler et al., 2010). However, there are also counter examples where gene flow occurs between different ploidy levels through at least partially fertile transitional cytotypes. This gene flow can facilitate heteroploid gene transfer, contribute to adaptation via adaptive introgression and even lead to the *de novo* formation of a new polyploid (Čertner et al., 2017; Kolář et al., 2017; Peskoller et al., 2021, reviewed by Brown et al., 2024; Bartolić et al., 2024). This might lead to a slowing down of the genomic separation of given cytotypes and hampering diversification and speciation as well. Besides, there are many cases of intraspecific variation in ploidy levels within the same species (e.g., *Hieracium* subgenus *Pilosella*, Suda et al., 2007; *Elettaria cardamomum*, Anjali et al., 2016; *Phragmites australis*, Wang et al., 2024), suggesting that auto-polyploidization does not necessarily drive rapid speciation. Regarding intraspecific genetic variation, ploidy level in populations may represent (i) different genetic groups (Balao et al., 2010), (ii) only a partial correspondence with the genetic clustering (Vanrell et al., 2024), or (iii) a complete mismatch indicating a lack of genetic differentiation among different ploidy levels within a species (Chumová et al., 2024; Kauai et al., 2024). Given the limited number of ancient polyploidization events inferred for plants, it seems that most of these frequent intraspecific polyploidy variations do not persist. Otherwise, we would observe many more ancient polyploidization events in extant species. This conclusion is congruent with recent polyploids showing often lower diversification rates than their diploid progenitors (Mayrose et al., 2010). This aligns with reports suggesting that polyploidization from a macroevolutionary viewpoint is an evolutionary “dead end” since polyploids exhibit higher rates of extinction than their diploid relatives (Arrigo and Barker, 2012; Mayrose et al., 2014; Shu et al., 2022). In contrast, there are also examples of autopolyploid cytotypes that have undergone speciation processes being ultimately recognized as independent species (Fernández et al., 2022). Other alternative views suggest that there is no significant association between shifts in diversification rates and ancient polyploidization (Landis et al., 2018).

The second line of theory emphasizes the role of CRs for recombination, whereby CRs become fixed through natural selection as they suppress recombination in locally advantageous groups of genes, known as supergenes (Ayala and Coluzzi, 2005). By acting as barrier loci, they create genomic regions that facilitate the maintenance of beneficial combinations of alleles within populations, even in the presence of gene flow and can ultimately promote reproductive isolation and speciation. The suppression-recombination model of chromosomal speciation predicts that chromosomal changes (inversions, dysploidy, deletions/insertions, duplications, and reciprocal translocations) that may affect recombination rates will occur before a cladogenetic event, where over time, locally adapted alleles accumulate, eventually resulting in a cladogenetic event. Under this model, we predict genome variation among populations of the same species, a variation that is indeed observed (Rieseberg, 2001a). Furthermore, the speciation process can occur concurrently with gene flow between different karyotypes, indicating that speciation is not an instantaneous process but often a rather gradual one influenced by ongoing evolutionary forces (Rieseberg, 2001a; Ravinet et al., 2017).

Both theoretical frameworks suggest that variation in CRs among populations facilitates reproductive isolation, thereby enhancing the potential for speciation (Lucek et al., 2023). In addition, these two models of chromosomal speciation are not mutually exclusive: some CRs may on one hand reduce gene flow between different karyotypes, resulting in partial hybrid dysfunction, and on the other hand suppress recombination. Together, these factors may eventually lead to cladogenesis. In this context, CRs that act as barriers to gene flow are predicted to occur either before the speciation process is complete or afterward, preventing interspecific gene flow, e.g. during secondary contact (Faria et al., 2011; Berdan et al., 2024). Finally, there are some CRs that are not or less important for speciation and are likely to become extinct over time (Lucek et al., 2023) or could be retained through a process similar to incomplete lineage sorting, where the origin of a CR predates the speciation event.

In a phylogenetic framework, the hybrid dysfunction type model is consistent with cladogenesis, whereas the suppression of recombination type model is more consistent with anagenesis (Lucek et al., 2022). However, only few phylogenetic approaches exist to model chromosomal evolution (Mayrose and Lysak, 2021). The joint modeling of chromosome evolution, speciation, and extinction is implemented in ChromoSSE (Freyman and Höhna, 2018). In this model, chromosomal changes may occur anagenetically (along the branches of the phylogeny) or cladogenetically (at the time of speciation). From the few examples for plants that implement this model (Freyman and Höhna, 2018; Valdés-Florido et al., 2023, 2024b; Tribble et al., 2025) a clear pattern emerges: the vast majority of chromosomal changes happen anagenetically, while only a small percentage occur cladogenetically. In summary, although chromosomal changes can occur around the speciation event, most changes occur between cladogenetic events. Additionally, it is thought that only a small percentage of chromosomal changes are able to survive through the filter of speciation, with most eventually being lost or becoming extinct.

Ultimately, chromosomal changes can indeed occur at the time of speciation, either initiating or reinforcing the process of cladogenesis (see more details in **Box 2**). In the hybrid dysfunction model, CRs such as polyploidy create reproductive barriers by reducing hybrid fitness. In contrast, the recombination suppression model proposes that chromosomal changes, like inversions or translocations, reduce recombination allowing locally adapted alleles to accumulate potentially driving speciation. In most cases, chromosomal changes do not persist long-term, as only a small percentage survive the speciation process, with most becoming extinct. Alternatively, CRs can also exist without affecting phenotypes or physiological functions, remaining neutral and simply persisting. Therefore, while CRs can contribute to speciation, the majority of these changes occur anagenetically, with relatively few occurring directly at the moment of cladogenesis.

### Box 2. From chromosomal assortative mating at population level to chromosomal cladogenesis across the phylogeny of holocentric true sedges

**Figure 4:**
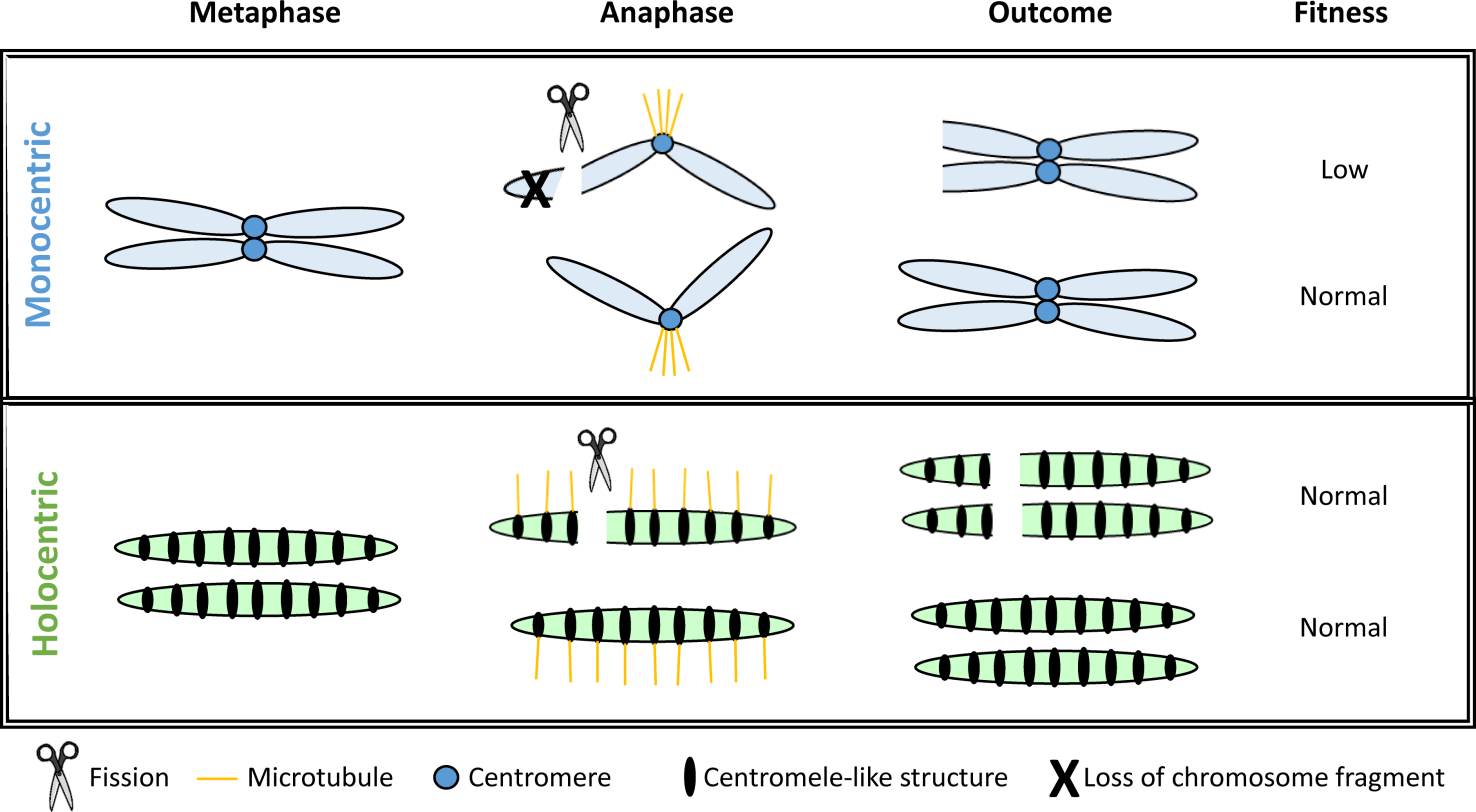
Modified from Lucek et al. (2022). Comparison of the outcomes of chromosome fission events during cell division for mono- and holocentric species.

The theory of chromosomal speciation primarily assumes that chromosomes are monocentric, meaning they contain a single centromeric region where all kinetochores are concentrated for spindle attachment during mitosis and meiosis (Escudero et al., 2016a). However, approximately 15-20% of extant eukaryotes, spanning 19 different animal and plant lineages, possess holocentric chromosomes. These chromosomes are characterized by holocentromeres: small, centromere-like regions dispersed along their entire chromosome length, rather than a single localized centromere (Escudero et al., 2016a). In holocentric species, segmental rearrangements may not lead to the same segregation issues during cell division as seen in monocentric species (Lucek et al., 2022). For example, in monocentric species, chromosomal fission can result in segments lacking a centromere, making them prone to loss during meiosis, while fusion events may create chromosomes with two centromeres, leading to segregation errors (**Figure 4**).

In monocentric species, when fission occurs, the chromosome fragment lacking a centromere is typically lost. In contrast, in holocentric species, fragmented chromosome sections can retain kinetochore function due to the distribution of centromere-like structures along the entire chromosome, allowing these fragments to be preserved (Lucek et al., 2022).

**Figure 5:**
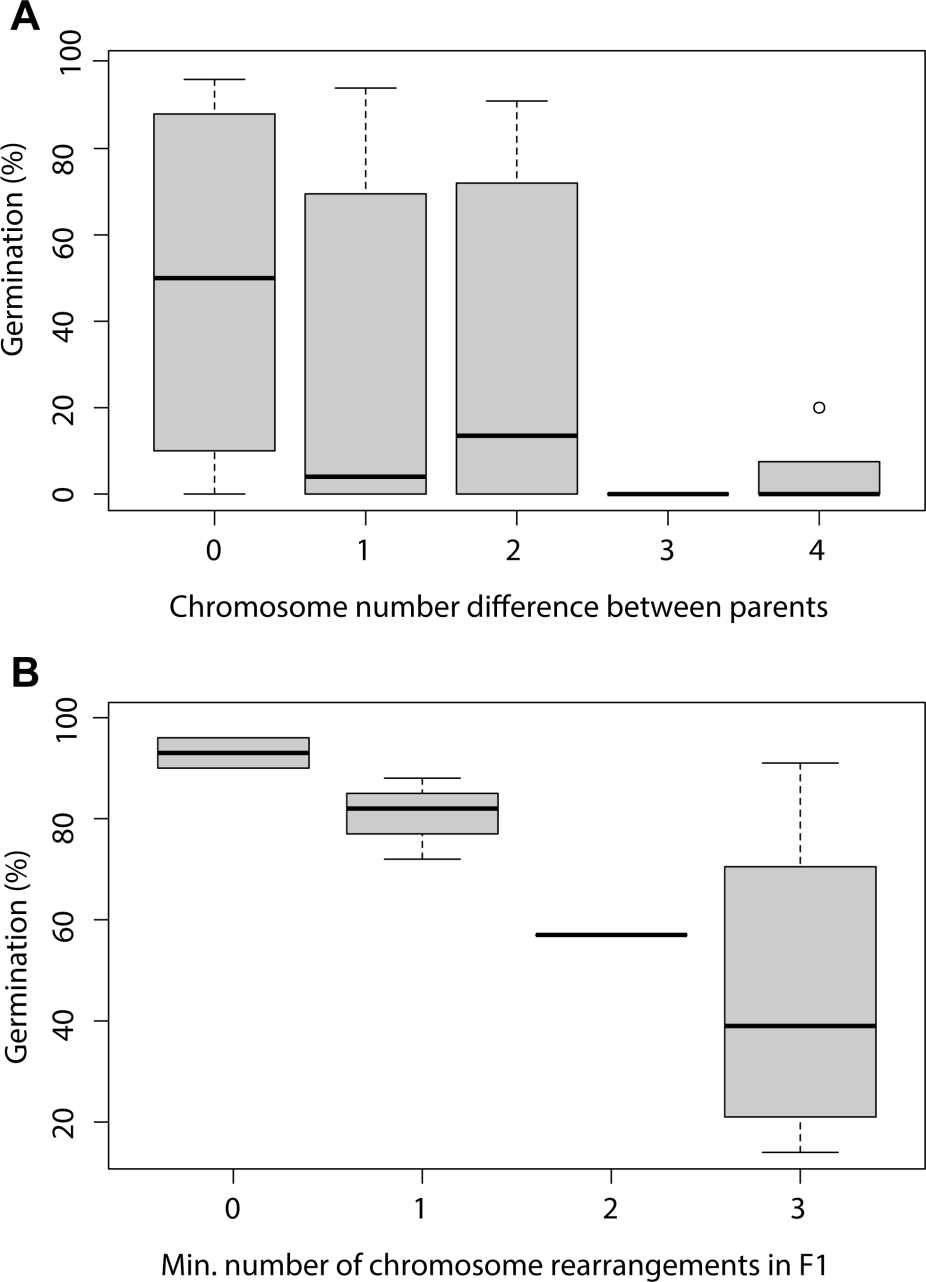
Modified from Escudero et al. (2016b). Boxplots showing seed germination percentages for **(A)** the offspring of artificial crosses between parent pairs with zero, one, two, three, and four chromosomal differences (N=33); and **(B)** F1 hybrids with zero, one, two, and three chromosomal irregularities (with the minimum number of irregularities considered) (N=11).

True sedges (*Carex*) belong to the holocentric sedge family Cyperaceae, one of the most diverse plant groups, comprising approximately 5,700 species (Larridon et al., 2021). The remarkable diversification of *Carex* is closely linked to its extensive variation in chromosome numbers (2*n* = 10–132), which has primarily evolved through chromosomal fusions and fissions, rather than polyploidization (Roalson, 2008). This unique evolutionary trajectory has positioned *Carex* as a model system for studying the dynamics of holocentric chromosomes and the mechanisms of chromosomal speciation, providing insights at both micro- and macroevolutionary levels. In *Carex* species, striking chromosome-number polymorphism is frequently observed, even within populations or individual plants (Whitkus, 1988; Luceño and Castroviejo, 1991; Escudero et al., 2013a, b; 2024). For example, *Carex scoparia* Schkuhr ex Willd. exhibits a range of 2*n* = 56 to 2*n* = 70 (Escudero et al., 2013b), *C. laevigata* Sm. ranges from 2*n* = 69 to 2n = 84 (Luceño and Castroviejo, 1991; Escudero et al., 2013a; Márquez-Corro et al., 2023), and *C. helodes* Link varies from 2*n* = 68 to 2*n* = 75 (Escudero et al., 2024). Experimental evidence from artificial crosses between cytotypes indicates that reproductive isolation intensifies as CRs accumulate, resulting in increasingly severe hybrid seed germination dysfunction (**Figure 5**; Escudero et al., 2016b; Whitkus, 1988). Nonetheless, individuals with differing chromosome numbers can often reproduce and exchange alleles—directly, if only minor CRs are involved, or indirectly (via individuals with intermediate karyotypes) in cases of major chromosomal differences—maintaining gene flow across chromosomal boundaries and species coherence (Escudero et al., 2013b). This suggests that while small chromosomal differences are insufficient to establish reproductive barriers, the accumulation of CRs can drive reproductive isolation over time (Hipp et al., 2009). The inferred isolation driven by gene flow and the accumulation of CRs in true sedges may significantly shape the genetic structure of populations. Hipp et al. (2010) found that both geographic distance and the number of karyotype rearrangements between populations influence the rate of gene flow in *C. scoparia*. A similar conclusion was reached at a finer evolutionary scale by Escudero et al. (2013a). Interestingly, this pattern also seems to apply at higher evolutionary levels, where the time to species coalescence is directly proportional to chromosomal variation within species in *Carex* sect. *Spirostachyae* (Drejer) L. H. Bailey (Escudero et al., 2010).

**Figure 6:**
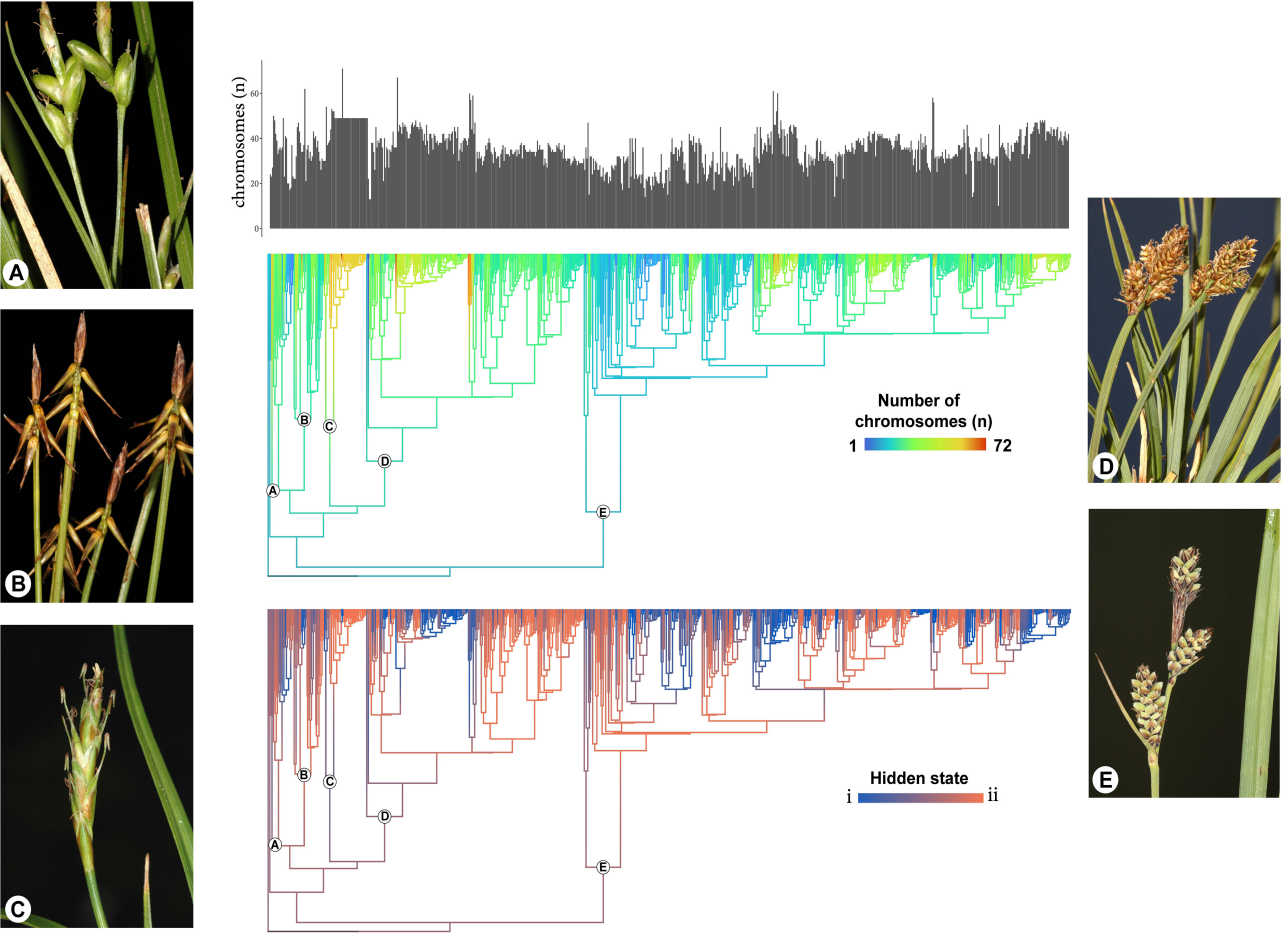
Modified from Tribble et al. (2025). Reconstruction of chromosome numbers and hidden states on the *Carex* phylogeny. At the top, the distribution of haploid chromosome numbers for all extant taxa included in the analysis. In the middle, the reconstructed evolution of chromosome numbers along phylogenetic branches. Warmer colors represent a higher number of chromosomes. At the bottom, the reconstructed evolution of the hidden states along phylogenetic branches. Blue color indicates strong statistical support for state i (cladogenesis driven by chromosomal changes), red color indicates strong support for state ii (cladogenesis is independent of chromosomal changes), and intermediary colors represent uncertainty in the estimates. Subgenera in phylogeny are labeled (**A** = *Psyllophorae*, **B** = *Euthyceras*, **C** = *Uncinia*, **D** = *Vignea*, and **E** = *Carex*). Photos display examples of species for each of the subgenera (**A** = *C. oedipostyla* Duval-Jouvé, **B** = *C. microglochin* Wahlenb., **C** = *C. meridensis* (Steyerm) J.R. Starr, **D** = *C. lucennoiberica* Maguilla and M. Escudero, and **E** = *C. adelostoma* V.I.Krecz.). Photo credits to M. Luceño.

The impact of dysploidy on *Carex* diversification has been previously explored using QuaSSE, which models chromosome number as a continuous trait (Márquez-Corro et al., 2021). Tribble et al. (2025) have been the first to jointly model chromosome number changes and diversification using a specialized model for chromosome evolution—the ChromoHiSSE model. This is a version of ChromoSSE (Freyman and Höhna, 2018) that accounts for hidden states, allowing the rates of chromosome number changes and their association with cladogenesis to vary across the phylogeny. Their results reveal an association between higher speciation rates and dysploidy in certain parts of the true sedge phylogeny, despite heterogeneity in the diversification process. In some clades, gains and losses in chromosome number drive diversification (hidden state i), while in other regions of the tree, these changes have the opposite effect (hidden state ii, see **Figure 6**). Furthermore, although dysploidy does not lead to higher speciation rates across the entire phylogeny, it strongly drives speciation in specific clades. Moreover, as indicated before, the vast majority of the CRs happen anagenetically along the branches of the phylogeny and only a small percentage of them are cladogenetic.

Tribble et al. (2025) proposed that the discrepancies in the impact of dysploidy on cladogenesis (with most changes happening anagenetically) may be linked to the unique nature of holocentric chromosomes. In this context, a single dysploidy event may not be sufficient on its own to induce reproductive isolation (Whitkus, 1988; Hipp et al., 2010; Escudero et al., 2016b; Lucek et al., 2022). However, the accumulation of CRs within a lineage over time could eventually establish a reproductive barrier, thereby driving speciation (Whitkus, 1988; Escudero et al., 2016b). This idea supports the hybrid dysfunction/recombination suppression model of chromosomal speciation, a central hypothesis discussed by Lucek et al. (2022). One possibility is that the accumulation of chromosomal changes eventually leads to reproductive isolation, with a “last straw” dysploidy event acting as the final trigger for speciation (the last-straw hypothesis in Tribble et al., 2025). Another possibility is that rearrangements in certain genomic regions are more stable than others, and the specific locations where fission or fusion occurs within the genome determine the evolutionary impact of dysploidy (Tribble et al., 2025).

## Q3: Do bursts of phenotype evolution, chromosome evolution, and speciation occur at the same time?

Changes in physical traits, in the chromosomes, and speciation can occur simultaneously in plants, but their interplay is complex and influenced by multiple factors. Intraspecific phenotypic and allelic changes are driven by natural selection, genetic drift or adaptation to local biotic and abiotic factors. Duplications, inversions, or translocations can result in gene expression changes that lead to significant phenotypic effects (Wray et al., 2003). As a consequence, these rearrangements can also lead to new linkage relationships or the formation of new genes. It is important to highlight that few breakpoints have been characterized for inversions with clear phenotypic effects (Hoffmann and Rieseberg, 2008; Guan et al., 2021; Chen et al., 2024). Previous studies have demonstrated that chromosomal inversions have putatively evolved as a response to environmental conditions because they were associated with morphological traits and showed increased fitness in adapted environments (Lowry and Willis, 2010; Lee et al., 2016). For instance, strong karyotype differences between closely related Mediterranean orchid species that also share pollinators have shown that CRs play an important role in reducing hybrid fitness and maintaining reproductive isolation (Cozzolino and Scopece, 2008). Otherwise, it is widely known that chromosomal deletions can have significant phenotypic consequences, since dominant alleles can be deleted, exposing recessive alleles in heterozygosity (Huettel et al., 2008). The phenotypic effects derived from other SVs have been less studied in plants. However, comparative genomic mapping has begun to facilitate their identification and annotation techniques have allowed the identification of possible SVs (including gene presence/absence and copy number variations) responsible for phenotypic traits (Huang and Rieseberg, 2020; Zhang et al., 2020). These kinds of studies have mainly focused on crops, identifying how SV impacts in genes with agronomic value (Yuan et al., 2021). Since crop gene pools are often derived from multiple species, sequencing and assembly efforts are put into all the species within the genus of interest. This has led to the development of super-pangenomes, which enable the detection of conserved and diverged genomic regions, as well as their frequencies within populations (Zhao et al., 2020). The relevance of pangenomics has grown significantly with the availability of high-quality genomes assemblies from multiple cultivars, especially in agriculturally important crops (Zhao et al., 2018).

Otherwise, WGD are common in plants, with extensive impacts on gene expression, cellular function, and organism phenotype. Polyploids can display differences in floral traits (Balao et al., 2011; McCarthy et al., 2015), chemical scents (Vereecken et al., 2010; Jersáková et al., 2010), and flowering phenology (Schranz and Osborn, 2000; Pegoraro et al., 2019). Such phenotypic changes evolved immediately after polyploidization, and it may have served to establish and stabilize novel cytotypes (Oswald and Nuismer, 2011; Clo and Kolář, 2021). Thus, polyploidy often results in reproductive isolation, leading to rapid speciation because new polyploid individuals may not be able to reproduce with their diploid progenitors. These new species frequently exhibit novel phenotypic traits as a consequence of changes in gene expression caused by the increased chromosome number (Chen, 2007; Balao et al., 2011; Basit and Lim, 2024). Phenotypic and morphological changes known to be induced by polyploidy are those related to variation in flower number and flowering time (Schranz and Osborn, 2000), plant structure, or alterations in plant physiology under stress tolerance (Cohen et al., 2013; Van de Peer et al., 2021; Turcotte et al., 2024). Polyploidy may for instance contribute to higher tolerance to nutrient-poor soils and resistance to stressful environments such as drought, cold or pathogens (Levin, 2002; Sader et al., 2019). In fact, a common phenomenon in polyploid species is the “gigas effect”, which results in increased cell sizes and overall plant features in comparison with their diploid parents (Stebbins, 1971; Soltis et al., 2014). In addition, CRs can generate genetic diversity through evolutionary changes, since bursts of lineage splitting in plants often result in adaptive radiation (Parent et al., 2020). In these cases, the accumulation of genomic changes leads to rapid phenotypic evolution promoted by genetic variation and accelerated evolution, particularly under changing environmental conditions. These phenotypic changes are not gradual but instead occur in bursts, often linked to speciation, which frequently occurs simultaneously. Consequently, long periods of evolutionary stasis could be interrupted by short and rapid bursts of evolutionary change linked to chromosomal events (Stebbins, 1971; Levin, 2002; Lysak et al., 2006). However, although most of intraspecific polyploidy variations do not persist over time (see above section), some studies have revealed that species with recent polyploid origins may undergo rapid speciation and significant phenotypic divergence (Fehrer et al., 2022). Floral evolution in the genus *Calochortus* Pursh (Liliaceae) represents a case of radiation, where selection for adaptation to diverse local habitats drives the specialization of flowers to various pollinators. This contrasts with adaptive radiation, which typically involves selection for specific pollinators within a single habitat (Patterson and Givnish, 2004). The ability to reproduce can also be directly linked to polyploidization, and typical patterns of cytotype distribution have been found in different studies (Krak et al., 2013; Valdés-Florido et al., 2024a). On the other hand, in isolated environments like Hawaiian Islands, plant species often display bursts of phenotypic diversity and chromosomal evolution as a consequence of rapid adaptation resulting in speciation events (Barrier et al., 1999; Bellinger et al., 2022). Thus, polyploidization is a process that assists speciation and diversification into new areas, being able to entail evolution of reproductive strategies. However, sometimes polyploidization could be related to the loss of a trait, such as it occurs with the loss of heterostyly for the two major families that present this trait (Primulaceae and Rubiaceae; Guggisberg et al., 2006; Naiki, 2012).

Aneuploidy has a very drastic impact on genetic dosage (Birchler et al., 2007; Birchler and Veitia, 2007) and is most commonly deleterious, while dysploidy is much more widespread with a high impact on plant evolution (Escudero et al., 2012). While dysploidy does not involve significant changes in DNA content, it can also have an impact on phenotype through structural rearrangements. At macroevolutionary scales, some studies highlight that gain or loss in the number of chromosomes can be associated with novel morphological features and influence diversification processes (Farminhão et al., 2021; Sader et al., 2019). For instance, recent research has shown that ascending dysploidy together with genome size expansion correlates both with larger flowers and higher diversification rates in the subgenus *Passiflora* L., suggesting a positive selection towards bigger genome sizes through morphological/ecological changes (Sader et al., 2019). The recurrent and parallel evolution of the same dysploid cytotype in the genus *Soldanella* L. has consistently resulted in speciation events (Slovák et al., 2023; Rurik et al., 2024). Similarly, Farminhão et al. (2021) found a correlation between dysploidy events and the evolution of leaflessness in the Dendrophylax-Microcoelia clade of angraecoids (Orchidaceae) with an eventful karyotypic history dominated by descending dysploidy, although the underlying mechanisms remain unexplored. No increases in net diversification rates could be related to chromosome number changes with the predominance of karyotypic stasis. However, species experiencing shifts in chromosome number appear to show parallel evolution of some phenotypic structures, leaflessness, and changes in floral color (Farminhão et al., 2021).

In summary, bursts of phenotypic and chromosomal evolution can occur simultaneously with speciation, but their relationship is complex due to the timing and interplay between these processes being highly dependent on evolutionary forces and environmental factors (see **Box 3** for a case study in the genus *Linum*). On the one hand, CRs, such as inversions or duplications, can lead to changes in traits like morphology, fitness, or reproductive isolation. On the other hand, polyploidy often leads to rapid phenotypic evolution and speciation due to changes in chromosome number, resulting in traits such as altered flowering times or increased environmental tolerance. Adaptive radiation often triggers rapid speciation and significant phenotypic divergence, but the persistence of these changes can vary since some chromosomal changes may become extinct over time, while others promote long-term diversification. Additionally, shifts in chromosome number, such as dysploidy or aneuploidy, can also contribute to phenotypic diversity and speciation, although their effects vary depending on the context.

### Box 3. Exploring biogeographic and ecological trait correlations in chromosome evolution: A case study in the genus Linum

**Figure 7:**
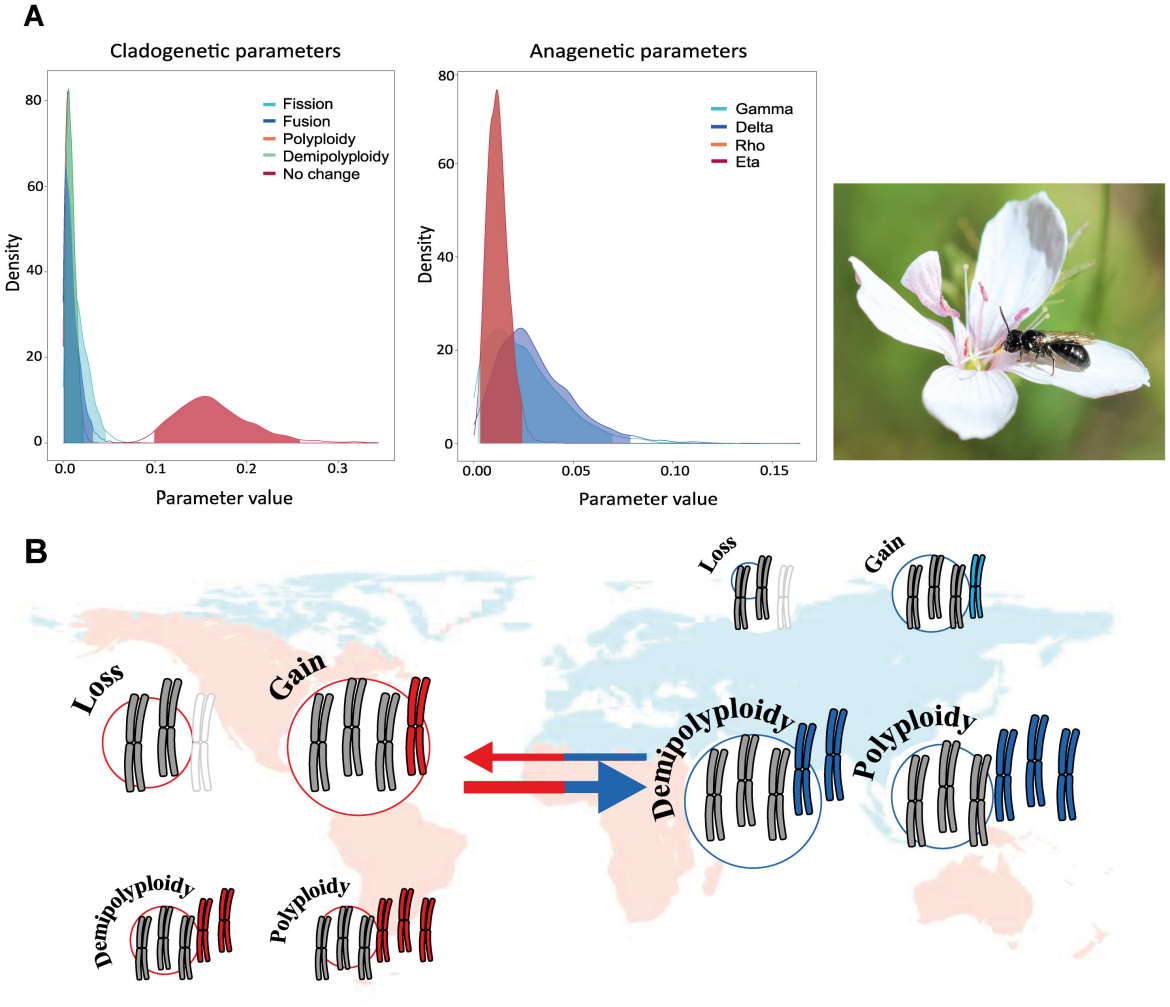
Modified from Valdés-Florido et al. (2023). **(A)** Posterior probability densities of the estimated clado- and anagenetic parameters using ChromoSSE in the *Linum* phylogeny. The x-axis displays the parameter value, and the y-axis indicates the posterior probability density of each value. The species in the photo is *L. tenuifolium* Schousb. Photo credits to B. Arroyo. Gamma represents the rate of chromosome gains, Delta the rate of chromosome losses, Rho the rate of polyploidization, and Eta corresponds to demipoliploidization, permiting the multiplication of the number of chromosomes by 1.5. **(B)** Correlation between chromosome number evolution and biogeography. Arrow and circle diameter is proportional to the rate.

The genus *Linum* L. (Linaceae) exhibits high rates of chromosomal evolution, primarily driven by polyploidy and dysploidy events. Despite this, only a limited number of chromosomal speciation events have been inferred across the whole phylogeny (Valdés-Florido et al., 2023). Specifically, five chromosomal speciation events were inferred, involving both ascending and descending dysploidy, along with two polyploid speciation events. These findings support the higher contribution of anagenetic events compared to cladogenetic ones (**Figure 7A**). In particular, species within the genus *Linum* are mostly diploid in the Palearctic region, being the ancestral area of distribution (Maguilla et al., 2021), whereas polyploid species are more common in regions outside this area. Rates of both ascending and descending dysploidy are higher in colonized areas, while polyploidization events are more frequent in the genus’ original distribution range (Valdés-Florido et al., 2023; **Figure 7B**). The model thus supports differing rates of chromosomal evolution between the source area and colonized regions. Interestingly, the elevated rates of dysploidy observed in colonized areas may be associated with *in situ* speciation events. This study also reveals a relationship between chromosome number and plant life history (annual vs. perennial). While most species are perennial, the rates of polyploidy are higher in annual species than in perennials, even though polyploidy has traditionally been associated with perennial life forms (Stebbins, 1971). This unexpected result may be explained by polyploidization events occurring in terminal short branches of some species. Besides, the woodiness and non-clonal nature of perennial species in *Linum* may account for this discrepancy, as polyploidy may not be associated with perenniality per se but rather with clonality (Van Drunen and Husband, 2019). Conversely, rates of descending dysploidy are significantly higher in perennial species.

## Q4: Does dispersal into new geographical areas or local environmental changes coincide with chromosome evolution and speciation?

One of the best-known drivers of speciation is geographic isolation, where populations of the same species become separated as a consequence of space in the presence or absence of geographical and ecological barriers (Dobzhansky, 1951; Coyne and Orr, 2004). Geographical isolation can provide the conditions for chromosome changes to accumulate either through drift or selection and without the diluting effects of gene flow from other populations. These chromosomal changes further enhance population differentiation in morphology, ecology, pre-and/or post-zygotic barriers, cumulatively or individually giving rise to reproductive isolation and eventually speciation (Levin, 2002; Scopece et al., 2010). This indicates that intrinsic postzygotic mechanisms may trigger polymorphism among allopatric conspecific plant populations. In contrast, a study found no links between chromosome transitions and the major diversification events associated with ecological events in the temperate grasses (Pooideae) (Pimentel et al., 2017). However, we should consider confounding factors in such macroevolutionary studies, such as the effect of undetected polyploidization followed by diploidization processes or genome sampling bias due to the use of few genetic markers, which does not negate the existence of chromosomal changes. In addition, chromosome evolution and cladogenetic processes were not modeled together which may lead to biased results if chromosomal changes are, in fact, affecting cladogenesis.

Changes in the environment (e.g., altitude, temperature) can also trigger adaptive responses through chromosome evolution (chromosomal inversion in adaptation; Huang and Rieseberg, 2020). Moreover, the occurrence of polyploidy in the tree of life also seems to correlate with periods of environmental change (Van de Peer et al., 2017). For instance, polyploidy can cause variation in plant functional traits and generate individuals that can adapt and exploit new environmental niches (Wan et al., 2020) and can facilitate adaptive response to harsh environmental conditions (Alix et al., 2017). Specifically, environmental stress has been proposed to foster the production of unreduced gametes, which are the main drivers of polyploidization in angiosperms (Bretagnolle and Thompson, 1995; Levin, 2002). The formation of diploid pollen grains has been promoted by low temperatures in the genera *Solanum* L. (Solanaceae)*, Datura* L. (Solanaceae)*, Oenothera* L. (Onagraceae), or *Epilobium* L. (Onagraceae) (e.g., McHale, 1983; Alsamir et al., 2021; Krakos et al., 2022). However, not only do low temperatures enhance the production of unreduced gametes, high temperature environments also have the potential to increase ploidy levels as seen in the genera *Rosa* L. (Rosaceae) (Pécrix et al., 2011; Crespel et al., 2015) and *Populus* L. (Salicaceae) (Wang et al., 2017). Other environmental factors, such as temperature fluctuations, low nutrient stress, or the presence of parasites and viruses have similarly been reported to promote the formation of unreduced gametes (Levin, 2002). These strategies are consistent with a broader adaptability and ecological tolerance and higher invasive potential of polyploids than their diploid relatives (Pandit et al., 2011; Te Beest et al., 2012; Marks et al., 2024).

Geographical and environmental pressures (or only one of them) may also occur simultaneously, driving chromosomal changes that indirectly promote speciation (Coyne and Orr, 2004). In addition, chromosome evolution has been strongly linked to biogeography in angiosperms (Rice et al., 2015), with polyploidization showing significant evolutionary implications, including the potential for range expansion (Te Beest et al., 2012; Soltis et al., 2015). For instance, in the genus *Panax* L. (Araliaceae) it has been demonstrated that the ancient and recent WGDs along with geographical and ecological isolations might have together contributed to the diversification of this genus, suggesting that distinct selection pressures appear to have acted during the genus’ evolutionary history (Shi et al., 2015). In the genus *Centaurium* (L.) Hill polyploid species may have an optimal climatic niche related to harsher environments (Valdés-Florido et al., 2024b; see **Box 4**). However, this cytotype adaptation is not always linked to speciation, as cytotypes can coexist within a single species as seen in the case of cryptic invasion of polyploid *Centaurea stoebe* L. expanding into the range of its diploid relative in Europe (Rosche et al., 2025).

Taking into consideration the above, geographic and environmental changes can trigger chromosome evolution, which can either coincide or accumulate post exposure together putatively driven by the severity of the changes experienced by plants. Geographic isolation is a key driver of this process, as it allows for chromosomal changes to undergo fixation in populations without gene flow. These chromosomal changes, including inversions and polyploidy, can contribute to further differentiation in traits, such as morphology and ecology (stress factors like temperature fluctuations or low nutrients availability), fostering reproductive isolation and speciation. In some cases, both geographic isolation and environmental pressures work together, while in other instances, environmental factors alone can influence chromosome evolution and diversification.

### Box 4. Chromosome evolution and climatic adaptation in Centaurium

**Figure 8:**
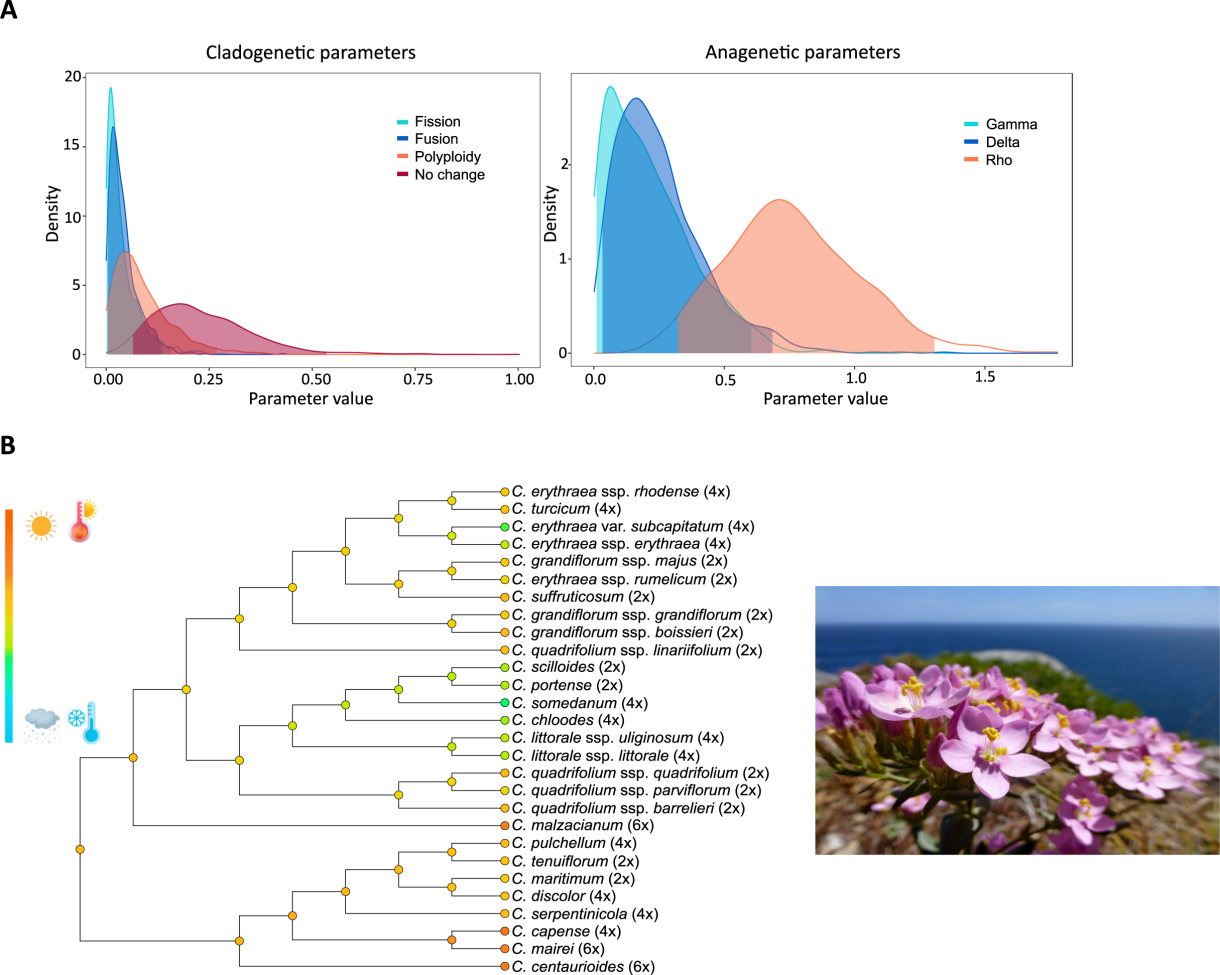
Modified from Valdés-Florido et al. (2024b). **(A)** Posterior probability densities of the estimated clado- and anagenetic parameters using ChromoSSE. The x-axis displays the parameter value, and the y-axis indicates the posterior probability density of each value. Gamma represents the rate of chromosome gains, Delta the rate of chromosome losses, and Rho the rate of polyploidization. **(B)** On the left climatic niche characterization of *Centaurium*. Colors in the nodes of the phylogeny correspond to the mean value of the climatic values used for the study. The species in the photo is *C*. *grandiflorum* ssp. *majus* (Hoffmanns. and Link) Díaz Lifante. Photo credits to S. Castro.

Some studies in the genus *Centaurium* (Gentianaceae) have examined the interplay between biogeography, climatic niche, and polyploid evolution. One of them revealed that diploid species primarily occupy the ancestral area at the Mediterranean Basin, while polyploids have successfully expanded into northern temperate regions as well as southern and eastern arid regions (Maguilla et al., 2021). Applying ChromoSSE to infer chromosome number evolution across the genus highlights several important patterns. Although a significant number of cladogenesis events are associated with polyploidization events, most cladogenetic events do not correspond to chromosomal changes. Anagenetic changes are associated with both dysploidy and polyploid events (**Figure 8A**; Valdés-Florido et al., 2024b). Polyploid speciation was inferred at both ancestral and more recent nodes and branches, while dysploidy events predominantly occur along terminal branches (Valdés-Florido et al., 2024b). Most transitions from diploid to tetraploid appear to be associated with transitions from drier, warmer to colder, wetter climatic niches, as well as the expansions from southern to northern distribution ranges (**Figure 8B**). In contrast, transitions leading to the hexaploids coincide with transitions from temperate to warmer and drier climatic niches at the southern distribution limit of the genus (**Figure 8B**; Maguilla et al., 2021; Valdés-Florido et al., 2024b). These findings suggest a strong link between polyploidization and climatic adaptation in the mostly Mediterranean *Centaurium* genus, with specific polyploid levels corresponding to distinct ecological niches and geographic distribution within its range. Although polyploidization itself does not necessarily drive dispersal within this genus, it appears to enhance the likelihood of establishment and persistence in newly colonized areas (Maguilla et al., 2021). Therefore, while geological barriers likely play a role in the speciation process of *Centaurium*, the observed pattern of niche expansion of polyploids may reduce competitive pressures and improve lineage survival.

## Q5: Do ecological interactions leave signatures on chromosome macroevolution?

Ecological interactions describe the diverse mechanisms through which organisms influence each other’s survival, reproduction success, and distribution within ecosystems. These interactions can occur between individuals of the same species (intraspecific) or between different species (interspecific) (Schoener, 1990), and play an important role in evolution, as they can act as selective forces during speciation (Thompson, 2009). From a microevolutionary perspective, empirical evidence supports the idea that ecological interactions have acted as selective forces on certain CRs (Burak et al., 2018). However, from a macroevolutionary point of view, there are not many cases where such interactions have left detectable signature in the speciation patterns among plant groups.

**Figure 9:**
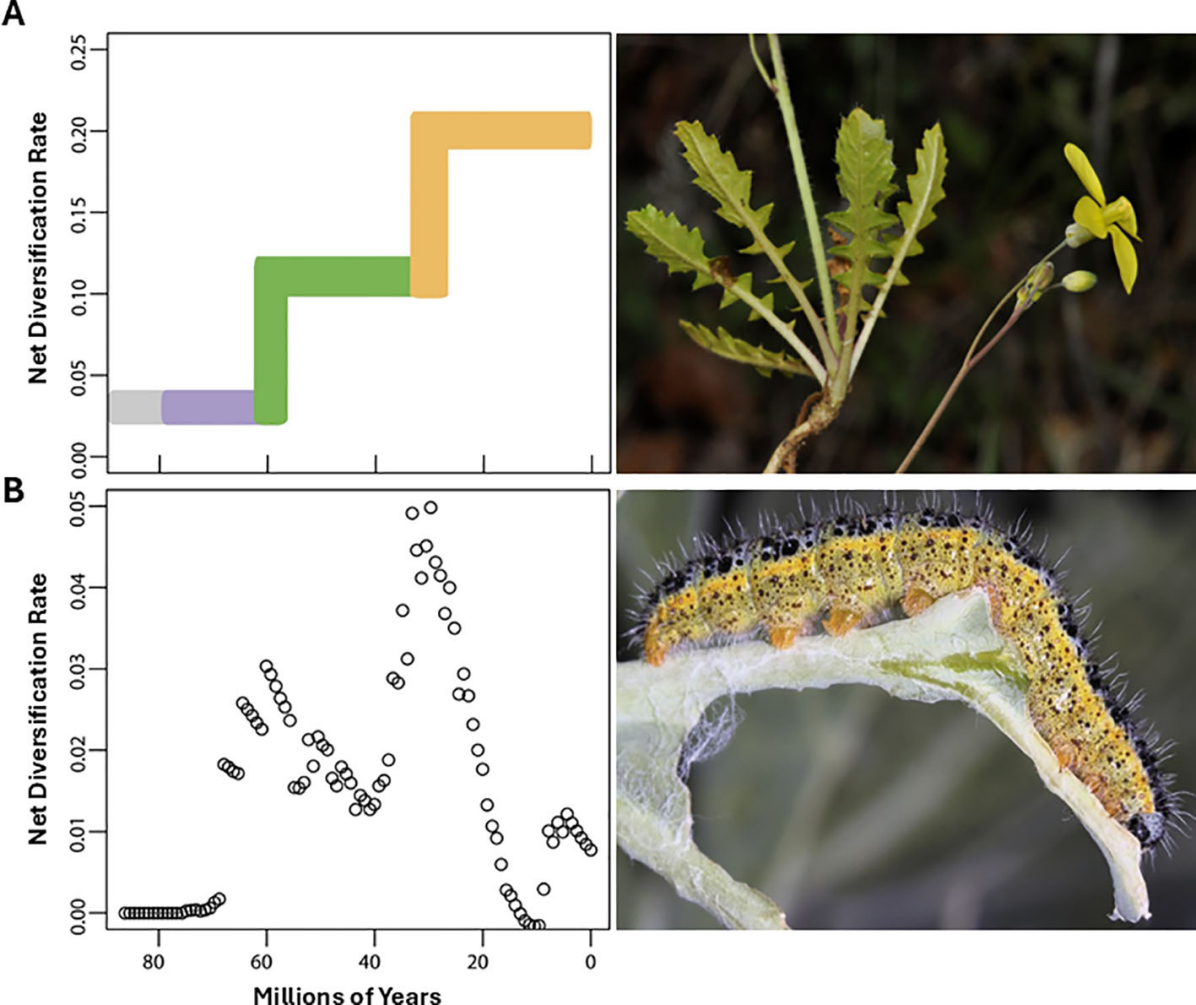
Modified from Edger et al. (2015). **(A)** Shifts in diversification rates during Brassicales Bromhead evolution. Colors indicate the emergence of indolic glucosinolates (purple), methionine derived glucosinolates (green), and novel structural elaborations in Brassicaceae Burnett lineage (orange). **(B)** Diversification of Pierinae butterflies during the same period. Time estimates are shown at the bottom. Photo credits: *Brassica barrelieri* (L.) Janka (top) by M. Luceño, and *Pieris brassicae* L. feeding on a *Brassica* species (bottom) by Edger et al.

Among the aforementioned CRs, polyploidy yet again, is the most widely studied in the context of ecological interactions (Thompson et al., 2004; Segraves and Anneberg, 2016). Polyploidization has the potential to create genetically isolated entities with divergent genetic and phenotypic traits that can shape the interactions of plants with other organisms, and likewise, these organisms can act as selective forces in stabilizing new polyploid races (Segraves and Anneberg, 2016). For species that rely on animal-mediated pollination, pollinators can contribute to the reproductive isolation of polyploids from their diploid relatives through assortative mating (Rezende et al., 2020). With the exception of the genus *Nicotiana* L. (Solanaceae), where pollinators have significantly influenced macroevolutionary patterns of speciation through floral color selection (McCarthy et al., 2015), clear examples of pollinators shaping diversification patterns in polyploid lineages are rare. In fact, the results of a current study, which examined whether neo-polyploidization in *Arabidopsis arenosa* (L.) Lawalrée led to changes in flower size that might influence pollinator behavior, found no evidence for assortative mating due to polyploidization. Instead, it was observed that polyploidization facilitated pollen exchange between different ploidy levels (Schmickl et al., 2024). Additionally, polyploids may evolve new defense mechanisms against herbivores, such as the production of new secondary chemical compounds (Orians, 2000; Edger et al., 2015; see more details in **Box 5**) or an expanded host range (Nuismer and Thompson, 2001; Arvanitis et al., 2010), which could also influence patterns of speciation in polyploid lineages and their diploid relatives. One of the most compelling examples of how key innovations responses to herbivory can shape macroevolutionary speciation patterns in plants through gene and WGD as described by Edger et al. (2015). In this scenario, the evolutionary arms race between members of the order Brassicales and pierid butterflies has played a significant role in driving the diversification rates of both groups (**Figure 9**). The evolutionary interplay between the two organismal groups has led to co-evolutionary dynamics, where plants evolve new chemical defenses against herbivory, while the butterflies develop mechanisms to overcome these defenses. Changes in the secondary chemistry of polyploids can also influence their interactions with other organisms, offering potential protection from parasites and pathogens (Burdon and Marshall, 1981; Vleugels et al., 2013) or disrupting relationships with mutualistic fungi, possibly resulting in fewer associations (Gundel et al., 2014; Franco et al., 2015). However, the underlying mechanisms remain complex and no documented cases have demonstrated a lasting impact on the macroevolutionary patterns of speciation in polyploid races. Lastly, an increase in genetic variability provided by polyploidization can enhance the environmental tolerance of polyploids, making them more competitive and prone to invasions (Pandit et al., 2011; Thébault et al., 2011; Te Beest et al., 2012; Cheng et al., 2021; Moura et al., 2021). This phenomenon is exemplified by certain species of the genus *Spartina* Schreb. (Poaceae), where meso- and neo-polyploid events have enabled them to be more competitive in stressful habitats by introducing novel regulatory patterns in gene expression (Giraud et al., 2021).

However, not only polyploidy but also dysploidy events may also have an impact in ecological interactions. A higher chromosome number may lead to higher recombination rates (Nijalingappa, 1974; Bell, 1982; Nokkala et al., 2004; Escudero et al., 2012). Higher recombination rates provide more evolutionary potential, which is advantageous in highly competitive communities with temporarily predictable environments (Koella, 1993; Escudero et al., 2013a; Escudero and Hipp, 2013). Holocentric chromosomes distribute centromeric activity along their length, unlike the single centromere of monocentric chromosomes (Márquez-Corro et al., 2018). This structure enables fragmented chromosomes to retain kinetochore function, reducing segregation errors during meiosis and potentially facilitating chromosomal fissions and fusions without compromising genome stability. In a highly competitive environment, this genetic flexibility can offer an advantage. Conversely, low rates of recombination should be positively selected in unstable and low competition communities where a pioneering strategy could be successful (Stebbins, 1958; Grant, 1958; Bell, 1982; Koella, 1993). Accordingly, in high competition environments, higher numbers of chromosomes are expected in holocentric species. This hypothesis has been tested at the microevolutionary level within *Carex laevigata* group populations (sect. *Spirostachyae*, Escudero et al., 2013a). The results indicated that chromosome numbers are indeed higher in lowland ancestral areas where competition is more intense. However, when the same hypothesis was extended to the whole genus *Carex*, the relationship between chromosome number and competition was less clear (Bell, 1982; Escudero et al., 2012). This indicates that other evolutionary or ecological factors could have shaped chromosome number evolution at a broader scale within this group.

Chromosomal rearrangements that do not involve changes in chromosome number can also play a role in adaptation by reducing recombination between favorable combinations of alleles (Kirkpatrick and Barton, 2006; Lowry and Willis, 2010). This reduced recombination contributes to speciation in a similar way - by suppressing recombination between local adapted alleles and those causing assortative mating (Trickett and Butlin, 1994). One of the examples is the differentiation of monkeyflowers (*Erythranthe guttata* G.L.Nesom, Phrymaceae) populations in two ecotypes, where a large inversion has been shown to affect the growth form (Lowry and Willis, 2010), herbivore resistance through secondary compounds synthesis (Kollar et al., 2024), as well as also causing assortative mating by allochrony in flowering time (Lowry and Willis, 2010). At a broader evolutionary scale, in this same genus, another inversion has been associated with differences in corolla length and flower color contributing to both prezygotic and postzygotic isolation of a sister species pair: *Erythranthe lewisii* (Pursh) G.L.Nesom and N.S.Fraga and *Erythranthe cardinalis* (Douglas ex Benth.) Spach (Fishman et al., 2013). Besides, Johnson et al. (2009) described SVs as having an effect on ecological interactions in plants at the macroevolutionary level. Under this scenario, some species from the Onagraceae family display Permanent Translocation Heterozygosity (PTH), a characteristic that prevents pairing of homologous chromosomes in meiosis and thus recombination. Moreover, most of these species are self-fertilizing, resulting in offspring that are genetically identical to the parent. The study found that species with PTH are more susceptible to generalist herbivores, suggesting that these may have reduced defenses against herbivory, likely due to their limited genetic variability.

Overall, ecological interactions leave a detectable signal on chromosome macroevolution, although the relationship is complex and not always straightforward. Evidence shows that ecological interactions, such as pollinator preferences, herbivory, and competition, can act as selective forces on CRs and influence speciation patterns in a microevolutionary and macroevolutionary scale.

### Box 5. Coevolutionary arms-race: gene and genome duplications driving diversification in plants and herbivores

One of the primary drivers of life’s diversity on Earth is coevolution between organisms that maintain close ecological interactions (Thompson, 2009). The mutual pressures that these organisms exert on each other can act as strong selective forces, driving the emergence of biodiversity. Moreover, as seen in this section, key innovations resulting from these selective pressures can be facilitated by CRs, creating new adaptive potential. Edger et al. (2015) shows how key innovations in response to herbivory, driven by gene and whole genome duplications, can influence macroevolutionary speciation patterns.

Plants from the order Brassicales can produce glucosinolates, a secondary metabolite which upon tissue damage are transformed into toxins, making them harmful to their main herbivores, the caterpillars of *Pierinae* Swainson butterflies. In contrast, these caterpillars have developed a mechanism to detoxify glucosinolates by utilizing a gene that encodes a nitrile-specifier protein (NSP), which converts these compounds into inert metabolites.

The acquisition of key innovations by plants and butterflies has unfolded in several stages. When Brassicales arose approximately 92 Mya, they could only synthesize glucosinolates from phenylalanine and branched-chain amino acids. The complexity of these chemical compounds diversified after a WGD approximately 77.5 Mya. This event enabled the production of indolic glucosinolates from tryptophan through the neofunctionalization of duplicated genes involved in glucosinolate synthesis. Adaptation of pierid butterflies to Brassicales occurred approximately 68 Mya and was facilitated by the evolution of glucosinolate detoxification. This key innovation significantly increased the diversification rate of the herbivores. Further complexity arose when additional gene duplications in the ancestors of Capparaceae and Cleomaceae plant families enabled the synthesis of methionine-derived glucosinolates, again via gene neofunctionalization, contributing to the diversification of Brassicales. However, the evolution of different copies of the NSP gene in Pierinae butterflies allowed them to adapt to these new compounds, as these copies developed functional differences against glucosinolates detoxification, which helped them overcome the plant defenses and enabled further colonization and diversification. The final phase of diversification took place around 32 Mya with the emergence of the Brassicaceae family. A subsequent WGD event enabled the origin and retention of genes involved in glucosinolate synthesis, driving the remarkable diversification of Brassicaceae. After their appearance, two different lineages of Pierinae butterflies (*Anthocharidini* Scudder and *Pierini* Swainson) colonized Brassicaceae. Both lineages utilized different copies of the NSP gene, each with functional adaptations to detoxify glucosinolates, and these colonization events coincided again with a significant increase in diversification rates.

## Q6: What is the role of hybridization-induced chromosomal changes in bridging microevolutionary processes with macroevolutionary patterns?

Hybridization serves as a critical evolutionary mechanism that plays a significant role in shaping diversification and speciation processes in plants (Soltis and Soltis, 2009; Taylor and Larson, 2019). It can play a creative role in species evolution, having both positive and negative effects, contributing to species evolution by triggering hybrid speciation (Abbott et al., 2013; Buerkle et al., 2000; Taylor and Larson, 2019), facilitating adaptive introgression (Suarez-Gonzalez et al., 2018), and even fueling adaptive radiations (Barrier et al., 1999; Mandáková et al., 2010b; Stankowski and Streisfeld, 2015). However, hybridization can also result in negative consequences, such as complete hybrid sterility, extensive introgression that merges previously separated gene pools, thereby hindering speciation and potentially leading to the extinction of parental taxa (Kearns et al., 2018; Slovák et al., 2023; Todesco et al., 2016). From a karyotypic perspective, the effects of hybridization depend on the compatibility of parental genomes, with ploidy level being a key factor. Hybridization can occur without changes in chromosome number (homoploid hybridization) or may involve WGD (allopolyploidization) (Ramsey and Schemske, 1998; Nieto Feliner et al., 2020; see a case study in **Box 6** for details on both mechanisms).

Allopolyploidization promotes speciation by integrating distinct parental genomes and establishing thus immediate reproductive barriers (Ramsey and Schemske, 1998; Pikaard, 2001; Mallet, 2007; Qiu et al., 2020). A major evolutionary advantage of allopolyploids is their increased heterozygosity, which confers hybrid vigor while overcoming some challenges of homoploid hybridization such as (sub)genomic incompatibility (Comai, 2005; Qiu et al., 2020). This can drive phenotypic changes and adaptations, potentially leading to further lineage diversification (Tayalé and Parisod, 2013; Mutti et al., 2017). However, allopolyploidization also presents remarkable challenges that can affect the viability and establishment of new allopolyploids, with potential negative implications for speciation. Newly formed allopolyploids face extrinsic challenges, such as population bottlenecks (Novikova et al., 2017) and competition with parental species (Fowler and Levin, 1984), as well as intrinsic challenges, including complications in chromosome segregation (Bomblies et al., 2016) and changes in the genome structure (Leitch and Leitch, 2008). As WGD significantly reduces or eliminates homeologous recombination in the hybrid genome, potential incompatibilities between divergent parental subgenomes cannot be effectively purged, which may pose significant challenges for newly formed allopolyploids (Rieseberg, 2001b; Qiu et al., 2020). As a result, the genomes of newly evolved allopolyploids must undergo continuous alterations at the genetic, epigenetic, transcriptomic, and proteomic levels during the early stages of establishment to achieve genomic stabilization (Chelaifa et al., 2010; Chester et al., 2012; Edger et al., 2017; Qiu et al., 2020). These genomic changes are typically absent in older allopolyploid lineages (Burns et al., 2021), indicating that structural and expression plasticity of genomes in newly formed allopolyploids is crucial for their stabilization and integration, with the timing of these changes varying among species (Wendel et al., 2018; Burns et al., 2021).

The association between allopolyploidy and diversification processes is frequently linked to biological traits; however, even greater attention has been given to adaptations within ecological and biogeographical contexts. One key aspect of establishment and subsequent evolution of allopolyploids is their niche breadth and shifts relative to their diploid progenitors. In contrast to previous assumptions (Stebbins, 1984), recent comparative studies have shown that ecological niche shifts in allopolyploids, relative to their diploid progenitors, are highly variable, exhibiting patterns of expansion, contraction, intermediacy, and novelty (Blaine Marchant et al., 2016; Parisod and Broennimann, 2016). Mata et al. (2023) further found no consistent differences in the distribution ranges or habitat types of allopolyploids in relation to extreme conditions; allopolyploid species do not necessarily occupy more extreme environments or broader geographic ranges compared to their diploid progenitors. The significant overlap observed between the niches and distribution ranges of allopolyploids and their progenitors suggests that these niches are largely shaped by the climatic and geographical characteristics of the parental species. However, biotic and microclimatic factors likely play a significant role in the establishment of allopolyploids (Blaine Marchant et al., 2016; Griffiths et al., 2019; Akiyama et al., 2020; Mata et al., 2023). Reevaluating the classical view that allopolyploids predominantly thrive in deglaciated temperate habitats (Stebbins, 1984) reveals contradictions considering recent findings. Indeed, an increasing number of studies emphasize the evolution of allopolyploids in the Mediterranean region, where they often exhibit restricted distribution ranges and specialized ecological niches (López-González et al., 2021; Šlenker et al., 2021; Kantor et al., 2023). It seems that the ecological dynamics of allopolyploids may be more complex than previously recognized, as their successful establishment in these environments suggests adaptive strategies that enable them to thrive despite often limited geographical distributions.

The critical question of whether allopolyploidy facilitates species diversification, especially at the macroevolutionary level through mechanisms such as species radiation, remains inadequately understood. Nevertheless, studies on allopolyploids in the tribe Microlepidieae (Brassicaceae) Al‑Shehbaz and approximately 50 taxa of *Nicotiana* sect. *Suaveolentes* Goodsp. (Solanaceae) suggest that all taxa in both groups are derived from a single allopolyploid ancestor, from which they diversified and radiated across the Southern Hemisphere (Clarkson et al., 2004; Kelly et al., 2013; Mandáková et al., 2017; Chase et al., 2023). In both cases, diploidization has led to the evolution of diverse dysploid lineages, facilitating further diversification processes (Mandáková et al., 2017; Chase et al., 2023). Similarly, a study by Tomlin et al. (2024) uncovered the allopolyploid origin of monophyletic Hawaiian mints (Lamiaceae), which are derived from North American ancestors of the genus *Stachys* L While the authors did not directly test the impact of allopolyploidy on the diversification and radiation of Hawaiian mints, they proposed that this allopolyploid ancestry could provide a genomic substrate for morphological differentiation within the lineage and potentially foster evolutionary radiation in the rapidly evolving Hawaiian landscape (Tomlin et al., 2024). In contrast, Estep et al. (2014) found that while one-third of species in the grass tribe Andropogoneae Dumort. (Poaceae) are allopolyploids, diversification did not precede the allopolyploidization event and does not correlate with subsequent speciation bursts. The emergence of Andropogoneae species in the Late Miocene coincides with the expansion of major C4 grasslands, and although allopolyploidy remains a significant mode of speciation within this tribe, its role in diversification is less clear. Furthermore, additional studies indicate that allopolyploidy may catalyze diversification and even radiation, although these hypotheses still need to be tested (Barrier et al., 1999; Julca et al., 2018).

In conclusion, allopolyploidy plays a significant role in rapid speciation by generating new species that exhibit hybrid vigor and enhanced ecological potential (Mallet, 2007). While it facilitates evolutionary diversification, the relationship between chromosomal evolution and ecological adaptation is variable and influenced by the ecological requirements of the progenitors. Although some studies suggest that allopolyploidy can trigger lineage diversification or rapid radiations, further comprehensive empirical investigations are needed to support these findings and allow for broader generalizations.

Homoploid hybrid speciation (HHS), in contrast to allopolyploid speciation, occurs without WGD, resulting in hybrid species that retain the same chromosome number as their parental species (Stebbins, 1958; Mallet, 2007; Soltis and Soltis, 2009; Abbott et al., 2010; Schumer et al., 2014). Unlike allopolyploidy, HHS does not immediately generate reproductive barriers. Instead, hybrid sterility, which restricts gene flow between hybrids and progenitors, must evolve through genetic incompatibilities (genic sterility) or CRs (chromosomal sterility) (Stebbins, 1958; Abbott et al., 2010; Yakimowski and Rieseberg, 2014). In the absence of reproductive barriers, homoploid hybrids are susceptible to backcrossing with their parental species, which can lead to introgression, the formation of hybrid zones, reinforcement, or even genetic assimilation (Soltis and Soltis, 2009; Todesco et al., 2016; Aguillon et al., 2022). An alternative mechanism for the stabilization and establishment of homoploid hybrid species involves extrinsic factors, such as spatial isolation from parental taxa, often accompanied by ecological divergence (Grant, 1981; Gross and Rieseberg, 2005; Abbott et al., 2010; Yakimowski and Rieseberg, 2014). Chromosomal rearrangements are widely recognized as a key factor for establishing intrinsic reproductive barriers between homoploid hybrids and their parental species, a phenomenon explained by the recombinational model of HHS (Stebbins, 1957; Grant, 1958, 1981; Buerkle et al., 2000). According to this model, parental species exhibit at least two independent CRs, resulting in reduced gamete viability in F1 hybrids due to heterozygosity. Subsequently, homozygous recombinants in the F2 generation may restore self-fertility while remaining incompatible with the parental species, potentially enabling sympatric speciation. This recombinational model demonstrates how hybrid lineages can achieve reproductive isolation from their parental species in sympatry, suggesting it as a likely pathway for initiating HHS. However, extrinsic reproductive barriers, such as ecological divergence between hybrids and parental species, may further influence the success of HHS (Buerkle et al., 2000).

Demonstrating HHS driven by chromosomal or genetic mechanisms, however, is not straightforward and requires a comprehensive investigation that integrates (cyto)genomic, ecological, and experimental approaches (Seehausen, 2004; Mallet, 2007; Schumer et al., 2014). This evidence includes reproductive isolation from parental species, documentation of past hybridization events, and confirmation that isolating mechanisms have emerged as a result of hybridization (Schumer et al., 2014). Therefore, evidence of HHS driven by CRs has only been documented in a limited number of plant systems (Rieseberg et al., 1995; Archibald et al., 2005; Wu and Tanksley, 2010; Yakimowski and Rieseberg, 2014; Ostevik et al., 2020). Nonetheless, the genus *Helianthus* L. (sunflowers) serves as the most iconic model system for understanding HHS, where CRs play a crucial role in establishing chromosomal sterility and driving speciation (Rieseberg et al., 1995; Ostevik et al., 2020; Todesco et al., 2020). The dynamic chromosomal evolution in sunflowers, driven by rearrangements, has facilitated rapid diversification, primarily through intrachromosomal inversions and interchromosomal translocations (Rieseberg et al., 1995; Ostevik et al., 2020). Both types of rearrangements are common in plant genome and karyotype evolution (Weiss-Schneeweiss and Schneeweiss, 2013), although intrachromosomal rearrangements tend to occur more frequently than interchromosomal ones in plant evolution (Wu and Tanksley, 2010; Ostevik et al., 2020). In addition, CRs that induce hybrid sterility appear to be strongly linked to an annual life strategy. These rearrangements are more frequent in annuals, which undergo more meiotic events per generation, thereby accelerating chromosomal mutation rates (Archibald et al., 2005; Owens and Rieseberg, 2014; Yakimowski and Rieseberg, 2014). While there is growing evidence supporting interspecific hybridization prior to adaptive radiations in both plants and animals (Seehausen, 2004; Meier et al., 2017; Stankowski and Streisfeld, 2015; Svardal et al., 2020; Skopalíková et al., 2023), documented cases of ancient homoploid hybridization preceding lineage diversification and radiation in plants remain scarce (Pease et al., 2016; Liu et al., 2017). Nevertheless, HHS occurs through genetic or chromosomal sterility. If the latter is involved, the extent to which CRs contribute to the homoploid speciation in the ancestors of these radiations remains unclear. In conclusion, the role of CRs in establishing reproductive barriers against closely related or more distantly related species during HHS-induced diversifications, and particularly radiation, remains largely unexplored. We propose that investigating the role of HHS and particularly the influence of CRs as a mechanism of speciation in plant diversification, presents significant opportunities for future research.

### Box 6. Evolutionary drivers and consequences of autopolyploidy vs. allopolyploidy: the case of Arabidopsis

*Arabidopsis* Heynh. is a leading model for plant genetics and physiology, primarily due to the well-studied species *A. thaliana* (L.) Heynh. However, the genus *Arabidopsis* is broader beyond the selfer *A. thaliana*, encompassing approximately six predominantly outcrossing diploid species of varying ecological niche and distribution area, with its center of diversity in Europe (Koch, 2019). In addition to sporadic reports of autopolyploid *A. thaliana* accessions (Bomblies and Madlung, 2014), there are two species encompassing established autotetraploid lineages: *A. arenosa* (Arnold et al., 2015; Kolář et al., 2016; Monnahan et al., 2019) and *A. lyrata* (L.) O'Kane & Al-Shehbaz (Schmickl et al., 2010; Bohutínská et al., 2024), and two allopolyploid species resulting from interspecific hybridization within the genus: *A. kamchatica* (Fisch. ex DC.) K. Shimizu & Kudoh (Paape et al., 2018; Kolesnikova et al., 2023) and *A. suecica* (Fr.) Norrl. (Novikova et al., 2017; Burns et al., 2021). The recent origin of both auto- and allopolyploids (Pleistocene; Novikova et al., 2018) within the same well-characterized genus has provided valuable opportunities to address general questions regarding the origins, post-WGD diversity dynamics, and the evolutionary significance of WGD and hybridization.

**Figure 10:**
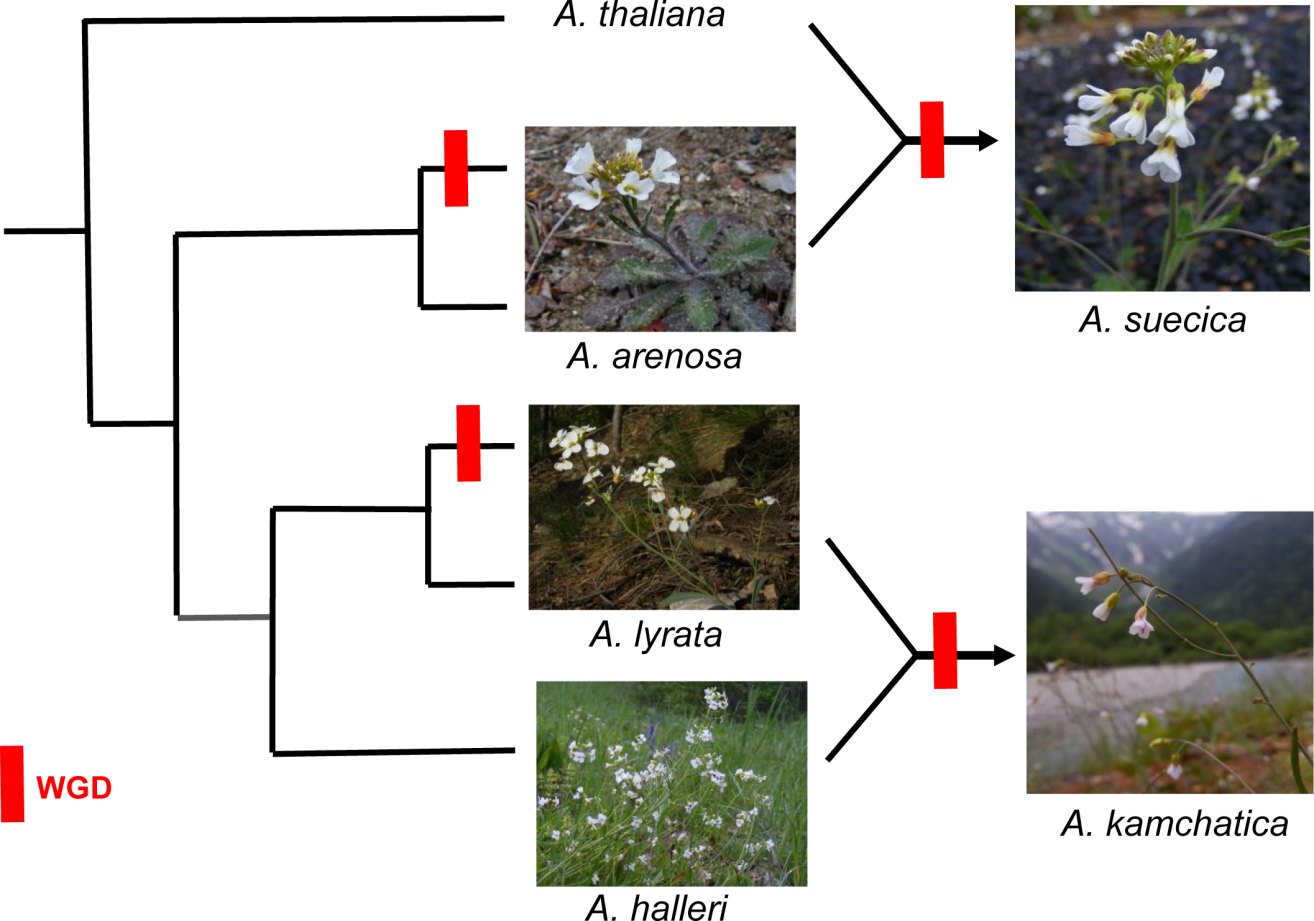
A tree showing the evolutionary history of six *Arabidopsis* species. The red bar labeled “WGD” indicates a Whole-Genome Duplication event that occurred in the lineage leading to *A. suecica* (Fr.) Norrl. and *A. kamchatica* (Fisch. ex DC.) K. Shimizu & Kudoh, and the polyploid lineages of *A. arenosa* (L.) Lawalrée and *A. lyrata* (L.) O’Kane & Al-Shehbaz. Photo credits to F. Kolář.

Interestingly, several distinct evolutionary features have emerged that differentiate these two types of polyploidy. Firstly, while autopolyploidy arises and remains exclusively in outcrossing lineages, allopolyploid origin is exclusively linked to a rapid transition toward selfing (Novikova et al., 2017, 2023; Monnahan et al., 2019; Kolesnikova et al., 2023). Secondly, post-WGD evolution appears to play an important role in shaping both adaptive and deleterious genetic variation in autopolyploids (Yant et al., 2013; Monnahan et al., 2019; Bohutínská et al., 2024; Vlček et al., 2025). In contrast, genetic variation inherited from diploid ancestors significantly contributes to the diversity of allopolyploids, underlying their patterns of breeding system (Novikova et al., 2018, 2023; Kolesnikova et al., 2023) and genome-wide diversity (Paape et al., 2018). Finally, the origin of polyploidy seems to be constrained by the availability of (pre)adaptive variation, which would enable rapid post-WGD adaptation to the challenges imposed by the transition to polyploidy (**Figure 10**). Some species have undergone none (*A. halleri* (L.) O'Kane & Al-Shehbaz and rare species) or few (single-WGD origin in *A. arenosa*; Arnold et al., 2015; Monnahan et al., 2019) WGD events, while others are prone to recurrent polyploidization (*A. lyrata*; Kolesnikova et al., 2023; Bohutínská et al., 2024; Scott et al., 2024). Moreover, the origin of allopolyploids seems to be constrained by the spatio-temporal availability of a self-compatible ancestral diploid lineage (Burns et al., 2021, 2024; Kolesnikova et al., 2023), which may enable further silencing of functional S-alleles from the outcrossing parent (Novikova et al., 2017). As expected in polyploid speciation, WGD imposes a strong postzygotic reproductive barrier between ploidies (Morgan et al., 2021). However, despite this triploid block, interploidy gene flow toward autotetraploids occurs in several natural ploidy contact zones (Jørgensen et al., 2011; Monnahan et al., 2019). Notably, WGD also opens up the possibility for further interspecific hybridization, including adaptive introgression, as seen in the case of post-WGD gene flow between autotetraploids of *A. arenosa* and *A. lyrata* (Marburger et al., 2019; Schmickl and Yant, 2021; Bohutínská et al., 2024; Scott et al., 2024). Thus, WGD in *Arabidopsis* not only acts as a speciation trigger (origin of polyploid lineages and species), but also as a factor dissolving species boundaries between previously reproductively isolated species (Lafon-Placette et al., 2017).

In summary, *Arabidopsis* shows that natural WGD transitions may go through multiple evolutionary trajectories, with their direction critically dependent on the mode of polyploid origin, ancestral variation, and the breeding system of the founding lineage(s). Investigating other systems in a comparative manner will provide insights into the generality of these findings and may further improve our understanding of possible transition cases between the two extreme modes of polyploidy, such as segmental allopolyploids.

## Discussion

### Challenges and outlooks in understanding CRs and evolution

Phylogenetic trees, which are used to reconstruct the timeline of cladogenetic events, are typically built using genetic divergence data, such as nucleotide substitution models, and/or morphological data. However, CRs and karyotypes are currently analyzed within pre-constructed phylogenetic trees, despite CRs being known to drive speciation through macroevolutionary dynamics. If insufficient time has passed since diversification, genetic divergence at the sequence level may not have accumulated sufficiently, leading to variability in estimates of divergence times depending on the phylogenetic datasets used. Even though large-scale CR and genomic architectural data were considered difficult to obtain a few years ago, their potential utility in reconstructing evolutionary relationships has long been recognized (Boore, 2006). Therefore, the inference of timeframes for cladogenetic events could be flawed if CRs were used as inputs for building phylogenetic trees. There may be a disconnection between sequence divergence, morphological divergence, and structural genomic divergence (including all forms of CRs), each of which could yield distinct phylogenetic outcomes.

Several methods have been developed to incorporate whole genome architecture data into phylogenetic reconstruction (Moret and Warnow, 2005; Lin et al., 2012). However, despite the increasing availability of whole genomes, these approaches have yet to gain widespread adoption in the field of evolutionary biology. It remains to be seen whether CRs will eventually be used to build phylogenies and/or resolve complex phylogenetic placement issues, and whether these methods will yield findings that differ from those produced by current sequence-based approaches.

### Unraveling missing pieces in CRs and evolution

Bridging the gap between micro- and macroevolution remains an outstanding goal in evolutionary biology. While macroevolutionary patterns and processes can be primarily inferred using indirect measures, future experimental work could shed light on the causality of the CRs as discussed in this review. The availability of long-read sequence data now allows us to establish pangenomes involving different scales, i.e. varieties, breeds, lineages to different species. Such pangenome approaches provide a comprehensive map of CR diversity at an unprecedented scope. Pan-genomes analyses have revealed that many CRs are polymorphic within species and are often linked to adaptation or domestication traits (e.g., Li et al., 2022; Jin et al., 2023; Jayakodi et al., 2024). Therefore, adopting a pan-genomic perspective is essential for a more robust assessment of the evolutionary significance of these CRs. Moreover, genome editing tools now enable direct testing of evolutionary implications of CRs for trait evolution, adaptation, and species diversification.

Chromosomal rearrangements and changes in ploidy are particularly important types of structural changes, as they not only affect genome structure at the genetic level but can also reshape the three-dimensional (3D) organization of the genome within a cell (Kumar et al., 2021). The 3D genome conformation is hierarchically packaged DNA at multiple levels to facilitate gene regulation and expression within a cell, with features such as chromatin loops and self-interacting genomic regions, known as Topologically Associated Domains (TADs), helping to organize interactions within chromosomes. Beyond these TADs, chromosomes are organized into distinct chromosomal territories, which are spatial compartments where both intra- and inter-chromosomal regions interact. Although TADs are not prominent in plants (commonly found in mammals and fruit flies; Dixon et al., 2012; Hou et al., 2012), TAD-like boundaries have been identified in plant genomes (Ouyang et al., 2020), indicating that 3D chromatin organization plays a role in genome function across different organisms. Furthermore, 3D genome chromatin states in plant genomes can be active, suppressed, or silenced through specific histone modifications, DNA methylation, and functioning of specific enzymes (Ouyang et al., 2020). Thus, alterations in the 3D genome structure, driven by CRs can impact gene expression, chromatin accessibility, and recombination patterns - processes that are directly relevant to species diversification. For instance, in some angiosperms chromosomes are arranged along a telomere to centromere axis (e.g. in common wheat *Triticum aestivum* L.; Hoencamp et al., 2021). By comparing the 3D genome structures of each subgenome in tetraploid cotton (*Gossypium hirsutum* L. and *G. barbadense* L.) with their respective diploid progenitors, it was found that genome polyploidization has influenced significant changes in genome organization (Wang et al., 2018). Specifically, polyploidization has driven the switching of active (A) and inactive (B) chromatin compartments and led to the reorganization of TADs (Pei et al., 2021). Thus, changes in the 3D genome can in turn affect gene expression, lead to a loss of chromatin accessibility, suppress recombination, and even may result in reproductive isolation (Álvarez-González et al., 2022). One mechanism through which the change in chromosomal 3D conformations can alter transcription is through the loss or gain of chromatin accessibility (Li et al., 2023). Together, these processes could lead to population differentiation, promote divergent adaptation and ultimately lead to speciation (Mohan et al., 2024). It suggests that CRs not only facilitate adaptation at the microevolutionary level but may also contribute to long-term macroevolutionary trends. Alternatively, sex chromosomes may fuse with autosomes, or new sex chromosomes may evolve, giving rise to reproductive strategies that can further facilitate CRs within the genome (Ming et al., 2011).

Thus, integrating insights from 3D genome organization with pangenomic studies of CRs provides a powerful approach to bridging micro- and macroevolutionary processes. By understanding how CRs and ploidy changes influence genome structure and function, we can begin to unravel the complex genetic mechanisms driving species diversification across different timescales. Furthermore, the interplay between CRs and epigenetic modifications, such as DNA methylation, adds another layer of complexity to genome function. These epigenetic changes, along with 3D genome conformation, may be playing a crucial role in regulating gene expression, recombination patterns, and, ultimately in shaping evolutionary species divergence.
